# Chinese Patent Medicine Liuweiwuling Tablet had Potent Inhibitory Effects on Both Wild-Type and Entecavir-Resistant Hepatitis B Virus (HBV) *in vitro* and Effectively Suppressed HBV Replication in Mouse Model

**DOI:** 10.3389/fphar.2021.756975

**Published:** 2021-10-27

**Authors:** Fei-lin Ge, Lan-lan Si, Yan Yang, Yuan-hua Li, Zhong-lin Lv, Wen-hui Liu, Hao Liao, Jun Wang, Jun Zou, Le Li, Hui Li, Zi-lin Zhang, Jia-bo Wang, Xue-chun Lu, Dong-ping Xu, Zhao-fang Bai, Yan Liu, Xiao-he Xiao

**Affiliations:** ^1^ School of Chinese Materia Medica, Beijing University of Chinese Medicine, Beijing, China; ^2^ Department of Infectious Diseases, The Fifth Medical Center of Chinese PLA General Hospital, Beijing, China; ^3^ Department of Liver Diseases, The Fifth Medical Center of Chinese PLA General Hospital, Beijing, China; ^4^ Department of Hematology, The Second Medical Center and National Clinical Research Center for Geriatric Diseases, Chinese PLA General Hospital, Beijing, China; ^5^ Department of Gastroenterology, The Second Medical Center and National Clinical Research Center for Geriatric Diseases, Chinese PLA General Hospital, Beijing, China

**Keywords:** hepatitis B virus, entecavir resistance, Chinese patent medicine, antiviral activity, active compounds

## Abstract

Liuweiwuling Tablet (LWWL) is a licensed Chinese patent medicine (approval number: Z20060238) included in the national health insurance for anti-inflammation of chronic HBV infection, whereas its anti-HBV effect remains clarification. The study aimed to clarify its antiviral effect and related mechanisms. HepG2.2.15 cells (wild-type HBV-replicating cells) and HepG2. A64 cells (entecavir-resistant HBV-replicating cells) were used for *in vitro* test. Hydrodynamic injection-mediated HBV-replicating mouse model was used for *in vivo* test. Active compounds and related mechanisms for antiviral effect of LWWL were analyzed using network pharmacology and transcriptomics. The inhibition rates of LWWL (0.8 mg/ml) on HBV DNA, HBsAg, and pgRNA were 57.06, 38.55, and 62.49% in HepG2.2.15 cells, and 51.57, 17.57, and 53.88% in HepG2. A64 cells, respectively. LWWL (2 g kg^−1^ d^−1^ for 4 weeks)-treated mice had 1.16 log_10_ IU/mL decrease of serum HBV DNA, and more than 50% decrease of serum HBsAg/HBeAg and hepatic HBsAg/HBcAg. Compared to tenofovir control, LWWL was less effective in suppressing HBV DNA but more effective in suppressing HBV antigens. Thirteen differentially-expressed genes were found in relation to HBV-host interaction and some of them were enriched in interferon (IFN)-β pathway in LWWL-treated HepG2.2.15 cells. CD3^+^CD4^+^ T-cell frequency and serum IFN-γ were significantly increased in LWWL-treated mice compared to LWWL-untreated mice. Among 26 compounds with potential anti-HBV effects that were predicted by network pharmacology, four compounds (quercetin, luteolin, wogonin, and kaempferol) were experimentally confirmed to have antiviral potency. In conclusion, LWWL had potent inhibitory effect on both wild-type and entecavir-resistant HBV, which might be associated with increasing IFN-β and IFN-γ production.

## Introduction

Hepatitis B virus (HBV) infection can cause chronic hepatitis B (CHB), increase occurrence risk of liver cirrhosis and hepatocellular carcinoma (HCC). An estimated 257 million individuals live with HBsAg positive, leading to more than 887,000 deaths annually (Revill et al., 2019). Two classes of anti-HBV agents, i.e., interferon (IFN) and nucleoside/nucleotide analogues (NAs), have been approved for treatment of HBV-infected diseases. So far, six NAs are licensed in China for the treatment of HBV-related diseases, including lamivudine (LAM), adefovir dipivoxil (ADV), telbivudine (LdT), entecavir (ETV), tenofovir disoproxil fumarate (TDF), and tenofovir alafenamide (TAF). These antiviral agents have brought great benefit for patients, while a major challenge is that HBV is hardly to be eliminated from patients with chronic HBV infection using current anti-HBV agents. In addition, HBV drug-resistance and adverse drug reactions (ADRs) are also factors influencing therapeutic efficacy. Therefore, it is still urgent to develop novel efficacious drugs and therapies to improve clinical cure of chronic hepatitis B (Wang et al., 2019; Seo et al., 2020; Helen et al., 2013; Kim et al., 2017).

A few of traditional medicine (TM) and related components were documented with anti-HBV effects, such as schisandrae chinensis fructus, salviae miltiorrhizae radix et rhizom, sophorae flavescentis radix, tsaoko fructus, wogonin, baicalein, and matrine (Liu et al., 2018; Si et al., 2019; Hepatobiliary Specialized Committee of China Association of Chinese Medicine and Liver Diseases Specialized Committee of China Medical Association of Minorities., 2018). Liuweiwuling Tablet (LWWL) is a drug approved by the China National Medical Products Administration (approval number: Z20060238) and it has been taken into the national health insurance. LWWL consists of six herbs, i.e., schisandrae chinensis fructus (*Wu Wei Zi* or WWZ), ligustri lucidi fructus (*Nv Zhen Zi* or NZZ), forsythiae fructus (*Lian Qiao* or LQ), curcumae rhizoma (*E Zhu* or EZ), field sowthistle herb (*Qu Mai Cai* or QMC), and ganoderma spore (*Ling Zhi Bao Zi Fen* or LZ). LWWL has good efficacy on anti-inflammation of chronic HBV infection (Hepatobiliary Specialized Committee of China Association of Chinese Medicine and Liver Diseases Specialized Committee of China Medical Association of Minorities., 2018; Hepatobiliary Specialized Committee of China Association of Chinese Medicine et al., 2020). In addition, a few of clinical reports indicated that LWWL combined with NAs could accelerate HBV DNA undetectability and normalization of alanine aminotransferase (ALT) compared to single NAs use (Zhao et al., 2016; Shangguan et al., 2016; Wang, 2014; Wang, 2020). However, the anti-HBV effects of LWWL and related mechanisms remains clarification. In light of the revelation, this study aimed to clarify anti-HBV effect and related mechanisms, and to identify major active compounds.

## Materials and Methods

### Cell Lines and Cytotoxicity Assay

Two HBV-replicating cell lines HepG2.2.15 and HepG2.A64 were employed in the study. HepG2.A64 as an ETV-resistant HBV-replicating cell line has been employed previously (Liu et al., 2016; Liu et al., 2018). Compared to HepG2.2.15 cells, HepG2.A64 cells generated comparable HBV DNA, higher HBsAg but lower HBeAg. The cytotoxicity of LWWL (Shibo Jindu, Zibo, China) and four compounds quercetin, luteolin, wogonin, and kaempferol (purchased from MedChemExpress Co., Ltd., Monmouth Junction, United States) on cells were analyzed using Cell Counting Kit-8 (Dojindo Laborarories, kyushu, Japan) according to the manufacturer’s instructions. The median cytotoxic concentration (CC_50_) was calculated. The molecular and cellular studies were carried out in Biosafety level-2 (BSL-2) laboratory at Center Laboratory, The Fifth Medical Center of Chinese PLA General Hospital. All manipulations were strictly conducted according to the instructions of the laboratories.

### Evaluating Anti-HBV Activity of LWWL in Cell Models

HepG2.2.15 cells and HepG2.A64 cells were respectively plated into 48-well culture plates (2 × 10^4^ cells/well). The cells in duplicate wells were treated with different concentrations of the drug (0, 0.1, 0.2, 0.4 and 0.8 mg/ml of LWWL, or 0, 0.2, 2, 20, 200 μmol/L of TDF, or selective concentrations of compounds identified from LWWL) for 5 days. Culture supernatants were harvested in 5 days for determining HBsAg and HBeAg levels by ELISA kits (Wantai Biological Pharmacy Enterprise Co., Ltd., Beijing, China), HBV pregenomic RNA (pgRNA) and HBV DNA levels were respectively determined by quantitative reverse-transcription PCR (qRT-PCR) and quantitative PCR (qPCR) assays as previously described (Ji et al., 2011; Liu et al., 2015; Wang et al., 2017). Half maximal inhibitory concentration (IC_50_) were calculated. The experiments were performed for three times independently.

### Establishment of HBV Mouse Model With Hydrodynamic Injection of Adeno-Associated Virus Plasmid (pAAV)-HBV1.2

The pAAV carrying 1.2-mer wild-type HBV genome was obtained from P.J. Chen (National Taiwan University, Taipei). C57BL/6 male mice weighted 20−22 g were injected into tail vein with 20 μg of pAAV-HBV1.2 plasmid in 2 ml PBS within 6–8 s. In 3 days, the mice were bled through orbit for monitoring HBsAg, HBeAg and HBV DNA levels.

### Evaluating Anti-HBV Activity of LWWL in the Mouse Model

The pAAV-HBV1.2-replicating mice were divided into four groups with six mice each group as follows: normal saline (NS) group, low-dose LWWL (1 g kg^−1^ d^−1^) group, high-dose LWWL (2 g kg^−1^ d^−1^) group, and TDF (63 mg kg^−1^ d^−1^) group. Intraperitoneal injection was conducted once a day for 4 weeks. The mice were bled weekly during treatment through orbit. The mice serum were harvested to measure HBV DNA, HBsAg, HBeAg, and IFN-γ using ELISA kits (Multi Sciences Co., Ltd., Hangzhou, China) according to the manufacturer’s instructions. Hepatic HBcAg and HBsAg were examined using immunohistochemical staining of paraffin-embedded tissue. Monoclonal mouse anti-HBs (MXB Biotechnologies, Fuzhou, China) and monoclonal mouse anti-HBc (Zhong Shan-Golden Bridge Biological Technology Co., Ltd., Beijing, China) were used for the examination. The animal study was conducted in BSL-2 laboratory at Animal Experimental Center, The Fifth Medical Center of Chinese PLA General Hospital. All manipulations were strictly conducted according to the instructions of the laboratories. The study protocol was approved by the Committee on the Ethics of Animal Experiments of The Fifth Medical Center of Chinese PLA General Hospital (Permit number: IACUC-2021-0009).

### Flow Cytometric Analysis

Effects of LWWL on splenic T cells activities were investigated for the high-dose LWWL mice (2 g^−1^ d^−1^). The mononuclear cells were isolated. CD3, CD4, CD8, and cell activation marker CD69 in mononuclear cells were visualized by fluorescent-labeled antibodies (Biolgend, California, United States) and subjected to LSRII flow cytometer. Data were analyzed using FlowJo software v10.

### Transcriptomics Analysis for Gene Expression Comparison

Total cell RNA were isolated from LWWL-treated, TDF-treated, and untreated HepG2.2.15 cells (named as LWWL group, TDF group and control group, respectively) and subjected to Agilent GeneChip (Shanghai Oebiotech Company, Shanghai, China) for transcriptomics analysis as previously described (Cheng et al., 2014). In brief, the differentially-expressed genes (DEGs) were analyzed using Kyoto Encyclopedia of Genes and Genomes (KEGG) database and gene ontology (GO) in the DAVID 6.8 (https://david.ncifcrf.gov/tools.jsp) to identify genes involved in HBV infection-related molecular interaction network. Transcriptomics data has been successfully deposited and is public. The accession number is GSE183509.

### RNA Quantification by qRT-PCR

The relative expression level of RNA of 24 DEGs was quantified using qRT-PCR, with 2^-△△CT^ method using β-actin as a reference control (Livak and Schmittgen, 2001). The primers are used in qPCR shown in Supplementary Table S1. TransStart Green RT-qPCR kit (Transgen Biotech Co., LTD., Beijing, China) was used to determining RNA level.

### Network Pharmacology Analysis for Active Compounds of LWWL and Targets of HBV

Firstly, the six herb names in LWWL were put into the traditional Chinese medicine systems pharmacology database (TCMSP) respectively to extract compounds of all herbs, the website address of TCMSP is http://tcmspw.com/. Furtherly, possible active compounds were filtered based on their corresponding pharmacodynamic parameters including drug-like (DL) and oral bioavailability (OB), and “DL ≥ 0.18, OB ≥ 30%” was used as screening conditions. Secondly, possible active compounds screened by OB and DL in LWWL were paired with potential target proteins based on TCMSP database, and the Uniprot database (https://www.uniprot.org/) was used to obtain the gene names of target proteins. Finally, Genecards database (https://www.genecards.org/) was used to extract HBV-related targets according to key words species “homo species” and “hepatitis B”.

### LWWL-Compounds-Target-HBV Network Analysis

The “LWWL-compounds-targets-HBV” interaction network was constructed using cytoscape software (Version 3.7.2) on the basis of the intersection of LWWL-related potential targets and HBV-related molecules. The value of degree was calculated to evaluate the anti-HBV potential of different compounds in LWWL. Targets of LWWL against HBV-related molecules through Cytoscape software analysis were placed in the DAVID 6.8 database for KEGG and GO analyses.

### Liquid Chromatograph-Mass Spectrometer Analysis of LWWL

The LWWL was qualitatively and quantitatively determined via LC-MS. The chromatographic conditions as below: Column, Phenomenex Kinetex 2.6u Bi-phenyl 100A, 50 × 3 mm; mobile phase A (water with 0.1% FA) and B (acetonitrile with 0.1% FA); elution program (0–0.5 min, 10% B; 0.5–4.0 min, 40% B; 4.0–9.0 min, 90% B; 12.0 min, 90% B; 12.0–15.0 min, 10% B). Flow rate was 0.4 ml/min; and injection volume was 5.0 μl. Electrospray positive ionization mode was used for analysis. The mass spectrometer was operated in positive mode with the main parameters set as follows: GS1 was 50 psi; GS2:50 psi; Curtain gas (N_2_) pressure was 35 psi; collision gas was nine psi; and capillary temperature was 550°C.

### Statistical Analysis

SPSS16.0 software was used for statistical analysis. The data are expressed as the mean ± standard deviation, and the experimental groups and the control group were analyzed by a *t*-test. Other data were analyzed by one-way analysis of variance (ANOVA). A *p* value <0.05 was considered statistically significant.

## Results

### Antiviral Effect of LWWL in Cell Models

**FIGURE 1:**
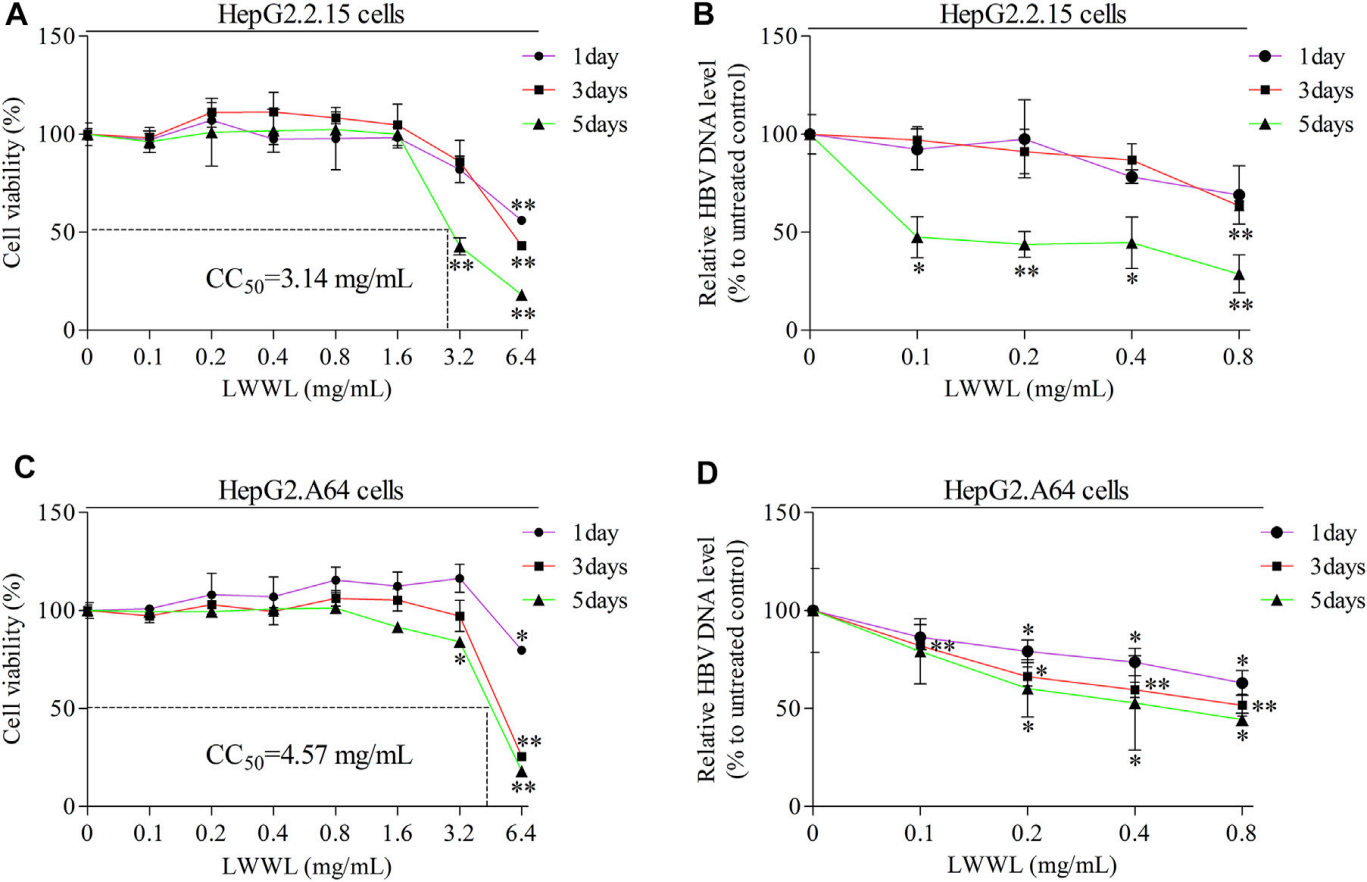
Evaluation of safe concentration and optimal effective time of LWWL against HBV in cell models. The safe concentration of LWWL (defined as that maintains ≥95% cell viability compared to drug-untreated control) were respectively evaluated in **(A)** HepG2.2.15 cells and **(C)** HepG2. A64 cells. The optimal effective time of LWWL against HBV (defined as the day-point with the strongest HBV DNA suppression during 5-days observation) were respectively evaluated in **(B)** HepG2.2.15 cells and **(D)** HepG2. A64 cells. Cell viability (A, C) and HBV DNA levels (B, D) between each of escalated concentrations of LWWL-treated groups and LWWL-untreated group are analyzed. * and ** represent *p* < 0.05 and *p* < 0.01 respectively in difference comparison between resultant *values* treated with each indicated LWWL concentration and the value treated with zero LWWL concentration using Student’s *t*-test. LWWL, Liuweiwuling Tablet; CC_50_, the median cytotoxic concentration against cultured cells.

Cell viability kept well when LWWL concentration was ≤0.8 mg/ml of LWWL. The CC_50_ values were 3.14 mg/ml and 4.57 mg/ml in both cell models (Figures 1A,C), respectively. Under the safe concentration, the antiviral effect of LWWL was evaluated on the first, third and fifth days respectively, and it was found that antiviral effect of LWWL was the best on day 5 of the treatment in both cell models (Figures 1B,D).

**FIGURE 2:**
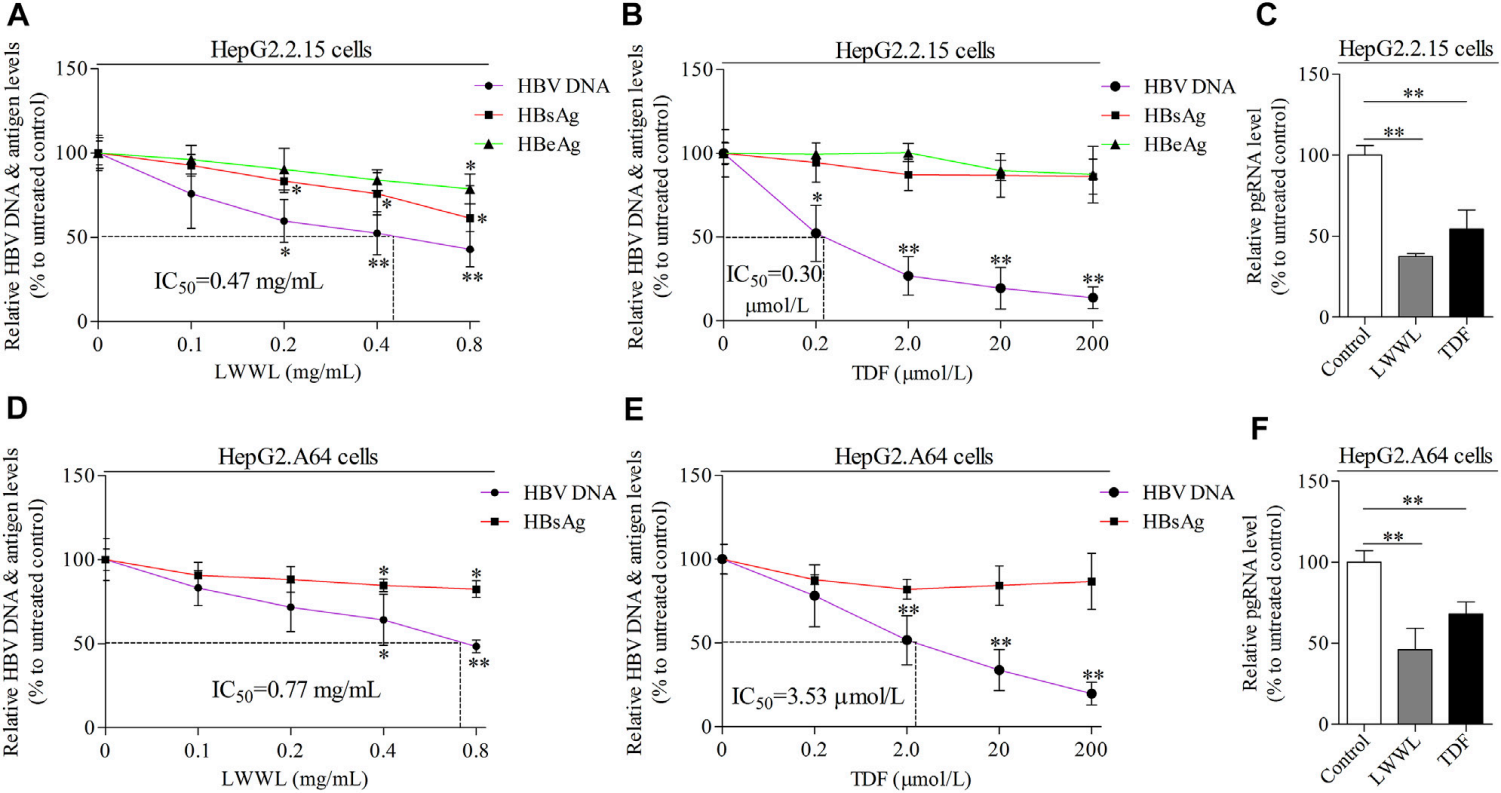
The effects of LWWL on HBV DNA/RNA and antigen in cell models. The inhibitory effects of LWWL on HBV DNA and supernatant HBsAg ± HBeAg were tested for both **(A)** in HepG2.2.15 cells and **(D)** in HepG2. A64 cells. The effects of TDF against HBV were also tested in **(B)** HepG2.2.15 cells and **(E)** HepG2. A64 cells. The effects of LWWL and TDF on supernatant pgRNA were also tested in **(C)** HepG2.2.15 cells and **(F)** HepG2. A64 cells. Dashed lines indicate IC_50_ of LWWL and TDF. * and ** represent *p* < 0.05 and *p* < 0.01 respectively in difference comparison between resultant *values* treated with each indicated concentration of LWWL (A, D) or TDF (B, E) and the value treated with zero concentration of LWWL or TDF. LWWL, Liuweiwuling Tablet; TDF, tenofovir disoproxil fumarate; pgRNA, pregenomic RNA; IC_50_, 50% maximal inhibitory concentration.

In wild-type HBV-replicating HepG2.2.15 cells, the inhibitory rates of LWWL (0.8 mg/ml) on HBV DNA, HBsAg, HBeAg and pgRNA were 57.06, 38.55, 21.26, and 62.49%, respectively (Figures 2A,C). By contrast, the inhibitory rates of TDF (200 μmol/L) on HBV DNA, HBsAg, HBeAg, and pgRNA were 86.18, 13.91, 12.66, and 45.55%, respectively (Figures 2B,C). In ETV-resistant HBV-replicating HepG2.A64 cells, the inhibitory rates of LWWL (0.8 mg/ml) on HBV DNA, HBsAg, and pgRNA were 51.57, 17.57, and 53.88%, respectively (Figures 2D,F). By contrast, the inhibitory rates of TDF (200 μmol/L) on HBV DNA, HBsAg, and pgRNA were 80.20, 13.26, and 31.93%, respectively (Figures 2E,F).

### Antiviral Effect of LWWL in Mouse Model

**FIGURE 3:**
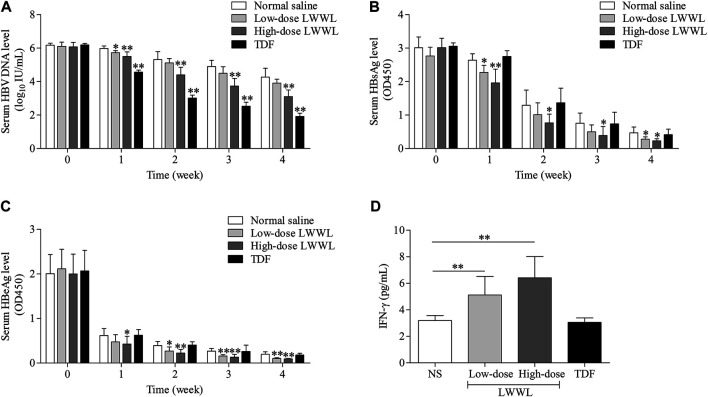
The impacts of LWWL on HBV DNA/antigen and IFN-γ of pAAV-HBV1.2 replication mice. The impacts of LWWL and TDF on serum **(A)** HBV DNA level, **(B)** HBsAg level, and **(C)** HBeAg level. **(D)** The impact of LWWL and TDF on serum IFN-γ level. * and ** represent *p* < 0.05 and *p* < 0.01 respectively in difference comparison between resultant *values* treated with each indicated concentration of LWWL or TDF and the value treated with NS. NS, normal saline; LWWL, Liuweiwuling Tablet; TDF, tenofovir disoproxil fumarate; interferon-γ, IFN-γ.

In 4-weeks treatment, serum HBV DNA levels were decreased 0.36, 1.16, and 2.35 log_10_ IU/ml in low-dose LWWL-, high-dose LWWL-, and TDF-treated mice compared to NS-treated mice. Serum HBeAg levels were decreased 47.47, 53.20, and 8.26%, and serum HBsAg levels were decreased 40.28, 50.77, and 11.53%, respectively post 4-weeks treatment (Figures 3A–C). Compared to NS-treated mice, serum IFN-γ levels of low-dose LWWL, high-dose LWWL, and TDF-treated mice had 1.60-, 2.01-, and 0.95-fold increases, respectively (Figure 3D).

**FIGURE 4:**
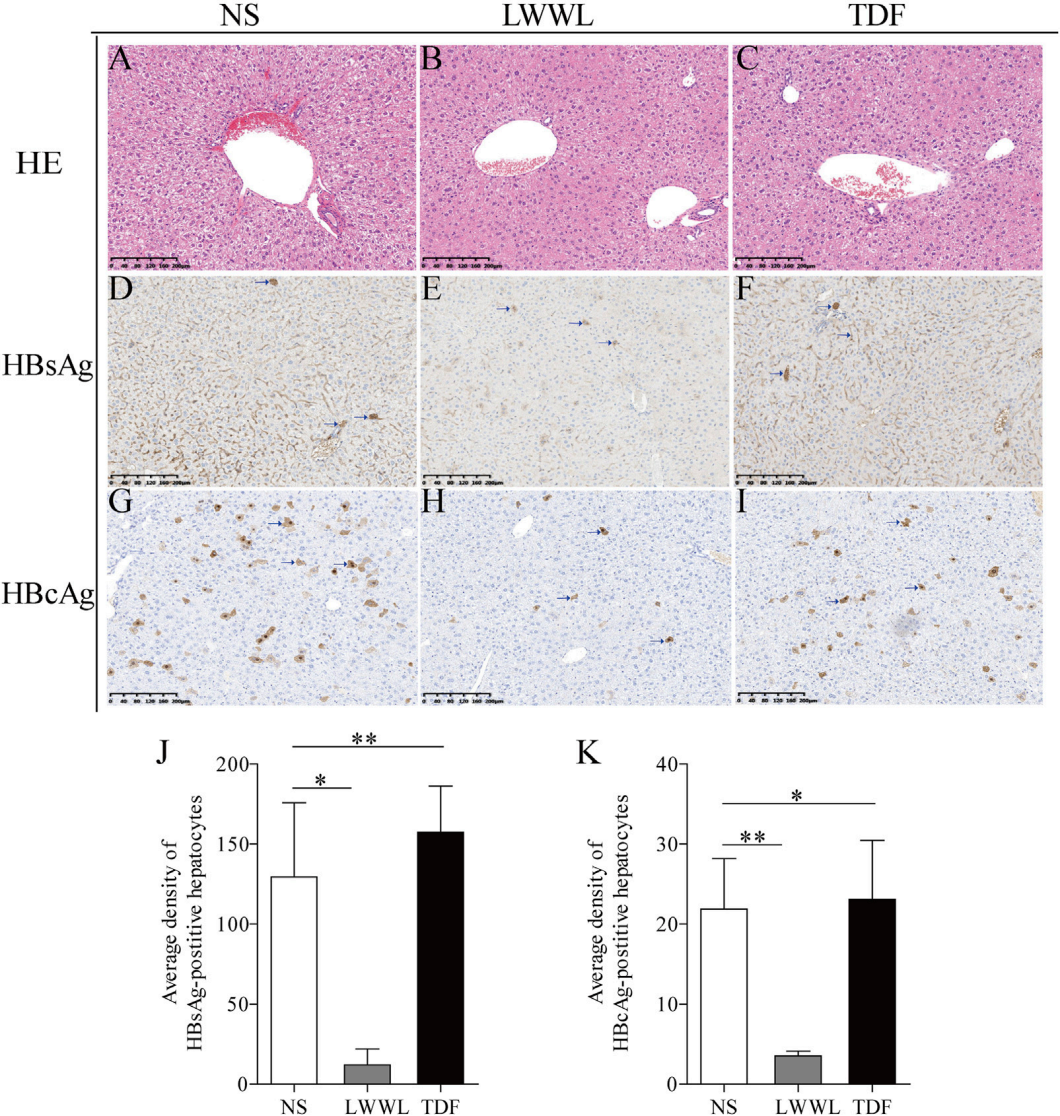
Histopathological examinations of LWWL-treated pAAV-HBV1.2 replication mice. HE was conducted for liver analysis of **(A)** NS-, **(B)** LWWL-, and **(C)** TDF-treated mice, respectively. Immunohistochemistric staining was conducted for HBsAg levels in liver of **(D)** NS-, **(E)** LWWL-, and **(F)** TDF-treated mice. Immunohistochemistric staining was conducted for HBcAg levels in liver of **(G)** NS-, **(H)** LWWL-, and **(I)** TDF-treated mice. Brown indicates the HBsAg- and HBcAg-positive hepatocytes. The quantitative analyses of **(J)** HBsAg expression and **(K)** HBcAg expression were also conducted in hepatocytes of NS-, LWWL- and TDF-treated mice. HE, Hematoxylin and eosin staining; NS, normal saline; LWWL, Liuweiwuling Tablet; TDF, tenofovir disoproxil fumarate. *p < 0.05, **p < 0.01.

The average densities of HBsAg-positive hepatocytes in the NS-, high-dose LWWL-, and TDF-treated mice were 129.48 ± 46.24, 12.02 ± 9.89, and 157.31 ± 29.05, respectively (Figures 4D–F). Average densities of HBcAg-positive hepatocytes of NS-treated, high-dose LWWL-treated, and TDF-treated mice were 21.88 ± 6.31, 3.54 ± 0.59, and 23.07 ± 7.40, respectively (Figures 4G–I). Statistical analysis showed that the number of the HBV antigen-positive hepatocytes was significantly decreased in LWWL-treated mice than in TDF-treated and NS-treated mice (Figures 4J,K).

### Effect of LWWL on Splenic T Cells Activation

**FIGURE 5:**
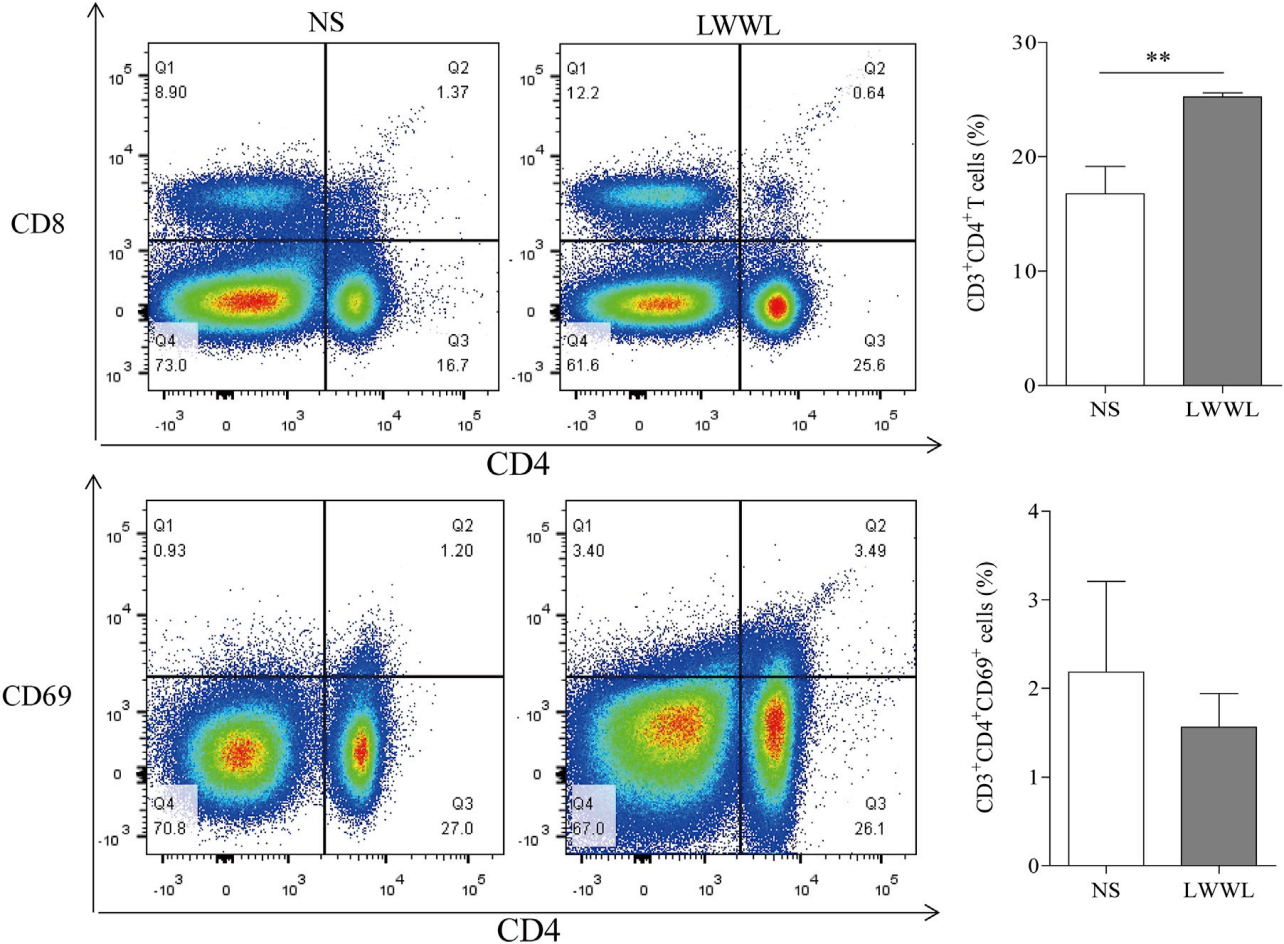
Effect of LWWL on splenic T cells of pAAV-HBV1.2 replication mice. Flow cytometry of the frequencies of CD4^+^ T cells **(A)** and CD4^+^CD69^+^ T-cell subsets **(C)** in gated CD3^+^ T-cell set of spleen lymphocytes are presented for representative pAAV-HBV1.2 replication mice from NS-treated and LWWL-treated groups. Statistical analysis of the frequencies of CD3^+^CD4^+^ T-cell subset **(B)** and CD3^+^CD4^+^CD69^+^ T-cell subset **(D)** was performed to see if there was significant difference between LWWL-treated group and NS-treated group. NS, normal saline; LWWL, Liuweiwuling Tablet. *p < 0.05, **p < 0.01.

The frequency of CD3^+^CD4^+^ T cells in high-dose LWWL-treated mice was significantly higher than that in NS-treated mice (17.10 ± 1.95% *vs* 25.43 ± 1.28%, *p* < 0.01) (Figures 5A,B); whereas no significant difference was observed for the frequencies of CD3^+^CD4^+^CD69^+^ T cells between LWWL-treated and NS-treated mice (1.97 ± 0.82 *vs* 1.63 ± 0.54%, *p* > 0.05) (Figures 5C,D).

### Differential Gene Expression in Transcription Level Based on Transcriptomics and qRT-PCR Verification

**FIGURE 6:**
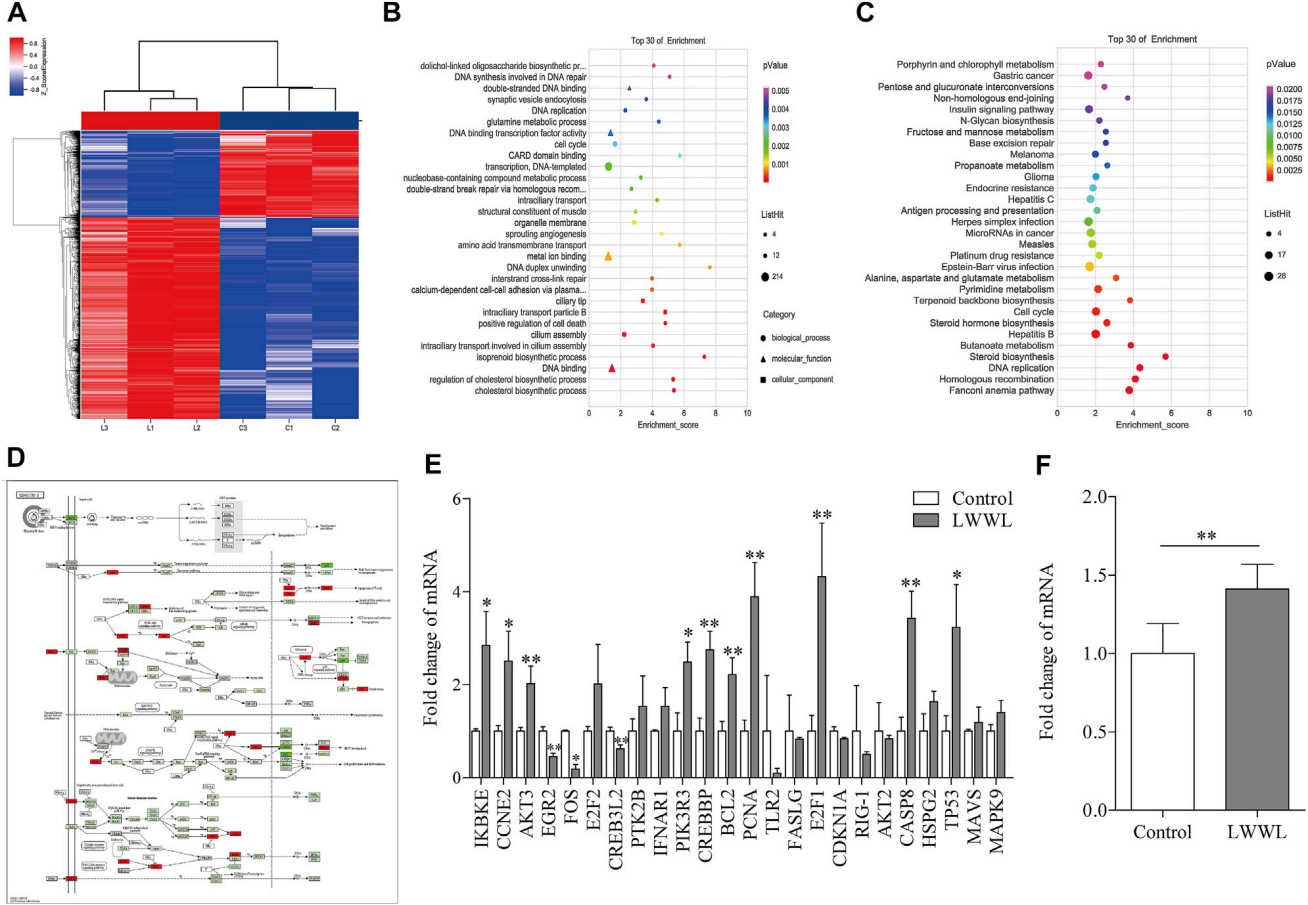
Analysis of target and pathway of LWWL against HBV based on transcriptomics in HepG2.2.15 cells. **(A)** Heat map of DEGs compared LWWL and control. Red region indicates significantly up-regulated genes of LWWL compared to control, and blue region indicates significantly down-regulated genes of LWWL compared to control. **(B)** The GO analysis of DEGs. **(C)** The KEGG pathway analysis of DEGs. **(D)** DEGs involved in HBV infection pathway by KEGG pathway enrichment analysis. Red boxes indicate significantly up-regulated genes of LWWL compared to control, and green boxes indicate significantly down-regulated genes of LWWL compared to control. **(E)** Validation of DEGs in HBV-related pathway by KEGG pathway enrichment analysis. **(F)** Validation of end effector molecules IFN-β in IFN-β pathway in HBV-related pathway. * and ** represent *p* < 0.05 and *p* < 0.01 respectively in difference comparison between resultant *values* treated with each indicated concentration of LWWL and the value treated with zero concentration of LWWL (control). LWWL, Liuweiwuling Tablet; interferon-β, IFN-β; DEGs, differentially-expressed genes; KEGG, Kyoto Encyclopedia of Genes and Genomes database; GO, gene ontology.

There were 2,074 up-regulated genes and 985 down-regulated genes in LWWL-treated HepG2.2.15 cells compared with untreated cells. Total of 24 DEGs was found to be involved in HBV-related pathway by KEGG pathway analysis in LWWL-treated cells compared to control group (Figures 6A–D).

Among them, 13 DEGs between LWWL group and control group were verified by RT-qPCR. Compared to control group, EGR2 and FOS expression in LWWL group were significantly lower, IKBKE, CCNE2, AKT3, CREB3L2, PIK3R3, CREBBP, BCL2, PCNA, E2F1, CASP8 and P53 expression in LWWL group were significantly higher (Figure 6E). In addition, IFN-β expression levels in LWWL group were significantly higher than that in control group (Figure 6F).

### Experimental Verification of Major Active Compounds of LWWL Associated with HBV Inhibition Based on Network Pharmacology Prediction

**FIGURE 7:**
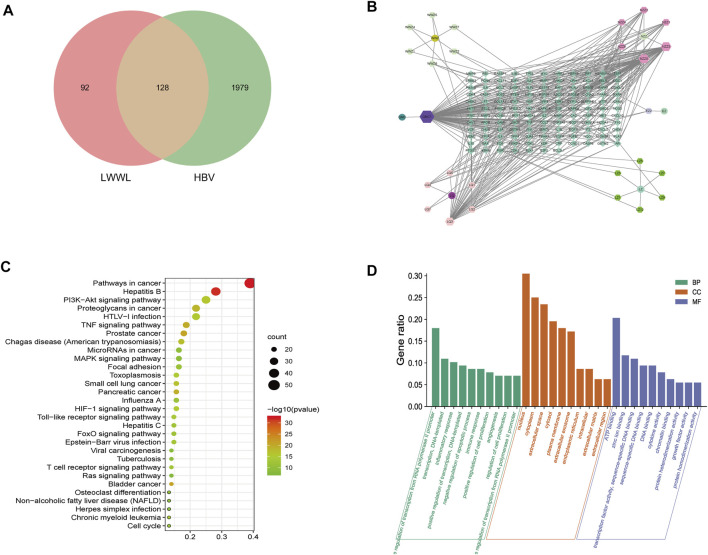
Analyses of active compounds and pathways of LWWL against HBV based on network pharmacology. **(A)** Veen diagram of targets in LWWL and HBV. **(B)** The LWWL-Compounds-Target-HBV network. Green diamond represents 128 putative targets of LWWL for the treatment of HBV. Cycle represents six herbs of LWWL including WWZ (Schisandrae chinensis fructus), NZZ (Ligustri lucidi fructus), EZ (Curcumae rhizoma), LQ (Forsythiae fructus), JMC (Field Sowthistle Herb), LZ (Ganoderma spore). Hexagon around cycle represents 26 active compounds of LWWL, and Table 1 for details. **(C)** The GO analysis of 128 targets of LWWL for the treatment of HBV. **(D)** The KEGG analysis of 128 targets of LWWL for the treatment of HBV. LWWL, Liuweiwuling Tablet. KEGG, Kyoto Encyclopedia of Genes and Genomes database; GO, gene ontology.

Total of 35 compounds contained in LWWL were subjected to network pharmacology prediction that contained 2107 HBV-related targets. As a result, 26 active compounds were found to be involved 128 HBV-related targets (Figure 7; Table 1).

**TABLE 1:** Prediction of anti-HBV components of LWWL based on network pharmacology.

Composition	Degree	OB%	DL%
quercetin	97	46.43	0.28
kaempferol	46	41.88	0.24
luteolin	45	36.16	0.25
wogonin	33	30.68	0.23
beta-sitosterol	13	36.91	0.75
bicuculline	12	69.67	0.88
Lucidumoside D_qt	12	54.41	0.47
taxifolin	9	57.84	0.27
Onjixanthone I	8	79.16	0.3
eriodictyol	7	71.79	0.24
(+)-pinoresinol monomethyl ether	6	53.08	0.57
(3R,4R)-3,4-bis[(3,4-dimethoxyphenyl)methyl]oxolan-2-one	6	52.3	0.48
Gomisin R	5	34.84	0.86
hederagenin	4	36.91	0.75
Angeloylgomisin O	3	31.97	0.85
Mairin	2	55.38	0.78
campesta-7,22E-dien-3beta-ol	2	43.51	0.72
ergosta-7,22E-dien-3beta-ol	2	43.51	0.72
ergosta-4,6,8(14),22-tetraene-3-one	2	48.32	0.75
ganoderal B	2	42.56	0.81
Lucialdehyde B	2	43.12	0.81
lucidone A	2	37.22	0.64
Gomisin-A	2	30.69	0.78
Wuweizisu C	2	46.27	0.84
Schizandrer B	2	30.71	0.83
Deoxyharringtonine	2	39.27	0.81

**FIGURE 8:**
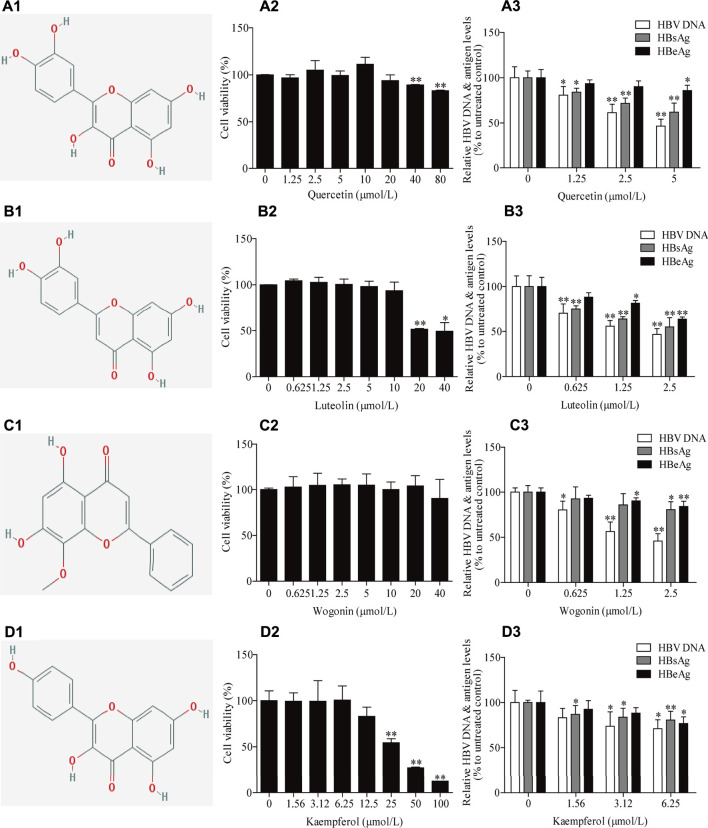
The inhibitory effects of four active compounds in LWWL on HBV DNA and antigens in cell models. The chemical structure of (A1) quercetin, (B1) luteolin, (C1) wogonin and (D1) kaempferol. Evaluation of safe concentration of (A2) quercetin, (B2) luteolin, (C2) wogonin, and (D2) kaempferol in HepG2.2.15 cells. The inhibitory effects of (A3) quercetin, (B3) luteolin, (C3) wogonin, and (D3) kaempferolon on HBV DNA, and supernatant HBsAg and HBeAg were observed in HepG2.2.15 cells. * and ** represent *p* < 0.05 and *p* < 0.01 respectively in difference comparison between resultant *values* treated with each indicated concentration of the tested compounds and the value treated with zero concentration of the tested compounds. LWWL, Liuweiwuling Tablet.

Among the 26 potential anti-HBV compounds, seven compounds (quercetin, kaempferol, luteolin, wogonin, beta-sitosterol, bicuculline, and lucidumoside D-qt) had >10 degree value were taken into further experimental analysis as a higher degree usually indicates a greater potential for anti-HBV activity. Among the seven compounds, quercetin, luteolin, wogonin, and kaempferol showed better anti-HBV effects. The molecular structures of the four active compounds are shown in Figure 8A1, B1, C1, D1. Cytotoxic testing showed the four compounds are shown in Figure 8A2, B2, C2, D2. The maximum concentrations with anti-HBV effects *in vitro* were 5.00 μmol/L for quercetin, 2.50 μmol/L for luteolin, 2.50 μmol/L for wogonin, and 6.25 μmol/L for kaempferol, respectively. As a result, the inhibitory rates on supernatant HBV DNA in HepG2.2.15 cells were 53.47, 53.28, 54.05, and 28.93% for quercetin, luteolin, wogonin, and kaempferol, respectively; and the inhibitory rates on HBsAg/HBeAg for the four compounds were 38.04%/14.25%, 45.00%/36.38%, 19.41%/15.95 and 19.35%/23.02%, respectively (Figure 8A3, B3, C3, D3). Furthermore, we have identified those four active compounds in LWWL based on LC-MS analysis (Table 2).

**TABLE 2:** Identification of the compounds of LWWL in chromatography.

Index	Component name	Formula	Precursor Mass	Found at Mass	Mass error (ppm)
1	luteolin	C_15_H_10_O_6_	287.055	287.0553	0.9
2	quercetin	C_15_H_10_O_7_	303.05	303.0504	1.5
3	wogonin	C_16_H_12_O_5_	285.076	285.076	1
4	kaempferol	C_15_H_10_O_6_	287.055	287.0554	1.5

## Discussion

The major drawback for current anti-HBV agents is that they do not effectively eliminate HBV from patients with chronic HBV infection. NAs are the most commonly-used antiviral agents. They effectively inhibit viral replication but has no direct suppressive effect on covalently closed circular DNA (cccDNA), and the suppressive effect is much weaker on HBV antigen expression than on HBV replication (Chevaliez et al., 2013). In addition, long-term use of NAs may induce HBV drug-resistance (Terrault et al., 2018). The life of HBV cycle and viral interplay with host involve multiple factors. Thus, agents reactive to multiple targets are required for effectively eliminating the virus (Wang, et al., 2021; Terrault et al., 2018). Because Chinese patent medicine contains multiple active components against multiple targets related to HBV proliferation, they may have a potential superior in playing multi-target synergistic antiviral effects (Liu et al., 2018; Hepatobiliary Specialized Committee of China Association of Chinese Medicine and Liver Diseases Specialized Committee of China Medical Association of Minorities., 2018). Clinical use of LWWL has shown that it is well efficacious on anti-inflammation of chronic HBV infection (Hepatobiliary Specialized Committee of China Association of Chinese Medicine et al., 2020). In addition, a meta-analysis documented that combination of LWWL with NAs (at least for 3 months) could increase the rate of HBV DNA undetectability (*OR* = 1.8–6.71, *p* < 0.05) and HBeAg loss (*OR* = 1.83–2.04, *p* < 0.05) compared to single use of NAs (He et al., 2017; Wang et al., 2020). So far, there is still lack of data on experimental anti-HBV effects and underlying mechanisms of LWWL.

Our study showed LWWL had anti-HBV effect both for wild-type and for ETV-resistant viruses. Compared to TDF control, LWWL was more efficient in suppressing HBV antigen levels, although it was less efficient in suppressing HBV DNA level. This antiviral effect was verified in HBV-replicating mouse. HBV antigen such as HBsAg is critical for HBV to establish immune tolerance, which could facilitate the persistence of HBV infection by suppressing host immunity through the regulation of IFN-related pathway (Jiang et al., 2014; Warner et al., 2020). LWWL had a better efficacy in suppression HBsAg production and this endow it potential to play a synergistic effect with NAs. In addition, compared to TDF, LWWL had a better effect on inhibiting HBV pgRNA of both wild-type and ETV-resistant HBV. As pgRNA is directly transcribed from cccDNA, it may more closely reflect cccDNA activity compared to the other viral markers (Wang et al., 2017).

Transcriptomics provides a novel and effective way to refine clues about complex mechanisms. Therefore, we used transcriptomics to analyze the potential mechanisms of LWWL against HBV. Transcriptomics analysis and qRT-PCR verification showed that there were 13 DEGs at transcription levels involved in HBV-related molecular pathways, mainly including P53, apoptosis, and IFN-β pathways. Further analysis showed that the expression level of effector IFN-β in IFN-β pathway were significantly higher in LWWL-treated HepG2.2.15 cells than that in LWWL-untreated cells. IFN-β has been verified in previous studies through activating pathogen-associated molecular patterns (such as TLRs, RIG-1, c-GAS)/IFN-β pathways (Yin et al., 2016; Cheng et al., 2017; Alexopoulou et al., 2020). Our results also showed that in HBV-replicating mouse model, LWWL treatment significantly increased the frequency of CD3^+^CD4^+^ T cells and serum IFN-γ production. IFN-γ is mainly generated by activated CD3^+^CD4^+^ T cells, and IFN-γ has been proved to be able to inhibit HBV replication through triggering intracellular antiviral pathways (Chokshi et al., 2014; Sang et al., 2017; Iannacone and Guidotti, 2021). We speculated that LWWL might play an anti-HBV role by enhancements of both IFN-β-mediated innate anti-HBV effect and IFN-γ-mediated acquired anti-HBV effect, whereas further studies are needed for the confirmation and elucidation of the context of the pathways.

Network pharmacology, developed in recent years, is an integration of bioinformatics and pharmacology by constructing the network of Chinese medicine-compounds-target-disease (Yang et al., 2018; Zhang et al., 2019). It has been proved to be a useful tool for predicting active compounds of Chinese medicine against certain diseases. Therefore, we firstly predicted the potential active compounds against HBV in LWWL based on network pharmacology, and then focused on the compounds with high degree that usually indicates a greater potential for anti-HBV activity. Out of the 26 compounds with potential anti-HBV effects that were predicted by network pharmacology, four compounds (quercetin, luteolin, wogonin, and kaempferol) were experimentally confirmed to have antiviral potency, which partly clarified the active compounds of LWWL against HBV, and also provides a reference for the new drug screening of HBV. Previous studies showed that luteolin had inhibitory effect on HBV replication through regulating HNF 4α expression (Bai et al., 2016), and quercetin could inhibit HBV DNA, HBsAg, and HBeAg levels *in vitro* but mechanisms remained clarification (Cheng et al., 2015). Our team reported that wogonin was one of major active compounds against HBV in Chinese herbal extracts Su-duxing, and wogonin had inhibitory effects on HBV cccDNA in addition to regular HBV DNA and antigens (Liu et al., 2018; Si et al., 2019). In this study, we confirmed that quercetin, luteolin, and wogonin in LWWL had anti-HBV effects, and found that kaempferol in LWWL was also an anti-HBV component. These enriched the knowledge on the anti-HBV effects of LWWL and provided a good start for elucidating the antiviral mechanisms of LWWL, although it is still a way to go to comprehensively elucidate the antiviral mechanisms of LWWL.

## Conclusion

In this study, we for the first time found that Chinese patent medicine LWWL could effectively suppress the activities of both wild-type and ETV-resistant HBV in cell models and the suppressive effects were superior to TDF on HBsAg expression. The antiviral effects were also verified in HBV-replicating mouse model. Our study suggested that LWWL against HBV might be associated with increasing IFN-β and IFN-γ productions. Four major active anti-HBV compounds from LWWL were firstly identified. These findings can provided new insights into the anti-HBV activities of LWWL, which may help optimize combination therapy of LWWL with current NAs and develop novel LWWL-derived anti-HBV agents.

## Data Availability

The datasets presented in this study can be found in online repositories. The names of the repository/repositories and accession number(s) can be found below: https://www.ncbi.nlm.nih.gov/geo/, GSE183509.

## References

[B1] Alexopoulou A. Vasilieva L. Karayiannis P. (2020). New Approaches to the Treatment of Chronic Hepatitis B. J. Clin. Med. 9, 1–23. 10.3390/jcm9103187 PMC7601587 33019573

[B2] Bai L. Nong Y. Shi Y. Liu M. Yan L. Shang J. (2016). Luteolin Inhibits Hepatitis B Virus Replication through Extracellular Signal-Regulated Kinase-Mediated Down-Regulation of Hepatocyte Nuclear Factor 4α Expression. Mol. Pharm. 13, 568–577. 10.1021/acs.molpharmaceut.5b00789 26656210

[B3] Cheng L. Hu W. Qiu B. Zhao J. Yu Y. Guan W. (2014). Generation of Neural Progenitor Cells by Chemical Cocktails and Hypoxia. Cell Res 24, 665–679. 10.1038/cr.2014.32 24638034 PMC4042166

[B4] Cheng Y. Ma J. Liu Y. Gao Q. Yan Y. Wang H. (2017). Chicken TBK1 Interacts with STING and Is Involved in IFN-β Signaling Regulation. Dev. Comp. Immunol. 77, 200–209. 10.1016/j.dci.2017.08.011 28837824

[B5] Cheng Z. Sun G. Guo W. Huang Y. Sun W. Zhao F. (2015). Inhibition of Hepatitis B Virus Replication by Quercetin in Human Hepatoma Cell Lines. Virol. Sin 30, 261–268. 10.1007/s12250-015-3584-5 26268473 PMC8200874

[B6] Chevaliez S. Hézode C. Bahrami S. Grare M. Pawlotsky J. M. (2013). Long-term Hepatitis B Surface Antigen (HBsAg) Kinetics during Nucleoside/nucleotide Analogue Therapy: Finite Treatment Duration Unlikely. J. Hepatol. 58, 676–683. 10.1111/jvh.1330610.1016/j.jhep.2012.11.039 23219442

[B7] Chokshi S. Cooksley H. Riva A. Phillips S. Williams R. Gaggar A. (2014). Identification of Serum Cytokine Profiles Associated with HBeAg Seroconversion Following Antiviral Treatment Interruption. Viral Immunol. 27, 235–244. 10.1089/vim.2014.0022 24797262

[B8] He X. Yang Y. X. Wen J. X. Zhao Y. L. Zhang L. Zhou H. Q. (2017). Systematic Review on Liuweiwuling Tablets Combined with Nucleotide Analogues in Treatment of Chronic Hepatitis B. Chin. Hospit Eval. Anal. Drug-use 17, 1–6. 10.14009/j.issn.1672-2124.2017.01.001

[B9] Helen K. Sabin C. A. Bruno L. Lene R. Worm S. W. Colette S. (2013). Antiretroviral Drug-Related Liver Mortality Among HIV-Positive Persons in the Absence of Hepatitis B or C Virus Coinfection: the Data Collection on Adverse Events of Anti-HIV Drugs Study. Clin. Infect. Dis. 56, 870–879. 10.1093/cid/cis91910.1093/cid/cit110 23090925 PMC3582358

[B10] Hepatobiliary Specialized Committee of China Association of Chinese Medicine and Liver Diseases Specialized Committee of China Medical Association of Minorities (2018). The Clinical Guidelines of Diagnosis and Treatment of Chronic Hepatitis B with Traditional Chinese Medicine. J. Clin. Hepatol. 34, 2520–2525. 10.3969/j.issn.1005-0264.2019.01.032

[B11] Hepatobiliary Specialized Committee of China Association of Chinese Medicine (2020). Chinese Patent Medicine Committee of China Association of Chinese Medicine and Clinical Pharmacy Professional Committee of Chinese Pharmaceutical AssociationClinical Application Expert Consensus of Treatment of Chronic Hepatitis B with Liuweiwuling Tablet. Chin. J. Int. Tradi West. Med. Liver Dis. 30, 482–485. 10.3969/j.issn.1005-0264.2020.05.034

[B12] Iannacone M. Guidotti L. G. (2021). Immunobiology and Pathogenesis of Hepatitis B Virus Infection. Nat. Rev. Immunol. 10.1038/s41577-021-00549-4 34002067

[B13] Ji D. Liu Y. Si L. L. Li L. Chen G. F. Xin S. J. (2011). Variable Influence of Mutational Patterns in Reverse-Transcriptase Domain on Replication Capacity of Hepatitis B Virus Isolates from Antiviral-Experienced Patients. Clin. Chim. Acta 412, 305–313. 10.1016/j.cca.2010.10.0210.1016/j.cca.2010.10.028 21056552

[B14] Jiang M. Broering R. Trippler M. Poggenpohl L. Fiedler M. Gerken G. (2014). Toll-like Receptor-Mediated Immune Responses Are Attenuated in the Presence of High Levels of Hepatitis B Virus Surface Antigen. J. Viral Hepat. 21, 860–872. 10.1111/jvh.12216 24498958

[B15] Kim J. H. Sinn D. H. Kang W. Gwak G. Y. Paik Y. H. Choi M. S. (2017). Low-level Viremia and the Increased Risk of Hepatocellular Carcinoma in Patients Receiving Entecavir Treatment. Hepatology 66, 335–343. 10.1002/hep.28916 28012257

[B16] Liu W. Song H. Chen Q. Xu C. Zhang W. Liu Y. (2016). Multidrug Resistance Protein 4 Is a Critical Protein Associated with the Antiviral Efficacy of Nucleos(t)ide Analogues. Liver Int. 36, 1284–1294. 10.1111/liv.13104 26931636

[B17] Liu Y. Li X. Xin S. Xu Z. Chen R. Yang J. (2015). The rtA181S Mutation of Hepatitis B Virus Primarily Confers Resistance to Adefovir Dipivoxil. J. Viral Hepat. 22, 328–334. 10.1111/jvh.12298 25132017

[B18] Liu Y. Yao W. Si L. Hou J. Wang J. Xu Z. (2018). Chinese Herbal Extract Su-Duxing Had Potent Inhibitory Effects on Both Wild-type and Entecavir-Resistant Hepatitis B Virus (HBV) *In Vitro* and Effectively Suppressed HBV Replication in Mouse Model. Antivir. Res 155, 39–47. 10.1016/j.antiviral.2018.04.017 29702120

[B19] Livak K. J. Schmittgen T. D. (2001). Analysis of Relative Gene Expression Data Using Real-Time Quantitative PCR and the 2(-Delta Delta C(T)) Method. Methods 25, 402–408. 10.1006/meth.2001.1262 11846609

[B20] Revill P. A. Chisari F. V. Block J. M. Dandri M. Gehring A. J. Guo H. (2019). A Global Scientific Strategy to Cure Hepatitis B. Lancet Gastroenterol. Hepatol. 4, 545–558. 10.1016/S2468-1253(19)30119-0 30981686 PMC6732795

[B21] Sang X. Wang R. Han Y. Zhang C. Shen H. Yang Z. (2017). T Cell-Aassociated Immunoregulation and Antiviral Effect of Oxymatrine in Hydrodynamic Injection HBV Mouse Model. Acta Pharm. Sin B 7, 311–318. CNKI:SUN:YXBY.0.2017-03-008. 10.1016/j.apsb.2017.02.005 28540167 PMC5430867

[B22] Seo J. W. Kim K. Jun K. I. Kang C. K. Moon S. M. Song K. H. (2020). Recovery of Tenofovir-Induced Nephrotoxicity Following Switch from Tenofovir Disoproxil Fumarate to Tenofovir Alafenamide in Human Immunodeficiency Virus-Positive Patients. Infect. Chemother. 52, 381–388. 10.3947/ic.2020.52.3.381 32757496 PMC7533205

[B23] Shangguan X. H. Zhao C. (2016). Observation on Curative Effect of Integrated Traditional Chinese and Western Medicine in the Treatment of Chronic Hepatitis B Patient. Henan Med. Resea 25, 1197–1198. 10.3969/j.issn.1004-437X.2016.07.021

[B24] Si L. L. Li L. Chen R. J. Bai Z. F. Niu M. Wang J. B. (2019). Combination Inhibitory Effect of Baicalein and Wogonin on Entecavir-Resistant HBV and the Optimal Ratio between the Two Components. Shandong Med. 59, 22–26. 10.3969/j.issn.1002-266X.2019.36.006

[B25] Terrault N. A. Lok A. S. F. Mcmahon B. J. Chang K. M. Hwang J. P. Jonas M. M. (2018). Update on Prevention, Diagnosis, and Treatment of Chronic Hepatitis B: AASLD 2018 Hepatitis B Guidance. Hepatology 67, 1560–1599. 10.1002/hep.29800 29405329 PMC5975958

[B26] Wang G. Liang P. Li P. Tan Y. H. Bonkovsky H. L. (2019). The Role of Traditional Chinese Medicines (TCM) and Other Complementary and Alternative Medicines (CAM) in the Management of Chronic Hepatitis B. Curr. Hepatol. Rep 18, 316–321. 10.1007/s11901-019-00480-2

[B27] Wang H. B. (2020). Clinical Efficacy of Liuweiwuling Tablets Combined with Entecavir in Patients with Chronic Hepatitis B. Health Vis. 11, 127.

[B28] Wang H. D. (2014). Clinical Study of Liuwei Wuling Tablets Combined with Entecavir in Treatment of Chronic Hepatitis B. Drug Clin. 29, 1023–1027. 10.7501/j.issn.1674-5515.2014.09.016

[B29] Wang J. Du M. Huang H. Chen R. Niu J. Jiang J. (2017). Reply to: "Serum HBV pgRNA as a Clinical Marker for cccDNA Activity": Consistent Loss of Serum HBV RNA Might Predict the "Para-Functional Cure" of Chronic Hepatitis B. J. Hepatol. 66, 462–463. 10.1016/j.jhep.2016.10.034 27826054

[B30] Wang Y. Y. Liu X. C. Piao R. L. Qin J. J. (2021). Research Advances in Anti-hepatitis B Virus Therapy Targeting Covalently Closed Circular DNA. J. Clin. Hepatol. 37, 1189–1192. 10.3969/j.issn.1001-5256.2021.05.044

[B31] Warner N. Locarnini S. Xu H. (2020). The Role of Hepatitis B Surface Antibodies in HBV Infection, Disease and Clearance. Future Virol. 15, 293–306. 10.2217/fvl-2019-0147

[B32] Yang Y. Yang K. Hao T. Zhu G. Ling R. Zhou X. (2018). Prediction of Molecular Mechanisms for LianXia NingXin Formula: a Network Pharmacology Study. Front. Physiol. 9, 489. 10.3389/fphys.2018.00489 29867541 PMC5952186

[B33] Yin J. W. Ping Huang M. Zhong B. (2016). Intrahepatic Toll-like Receptor 3 in Chronic HBV Infection Subjects: Asymptomatic Carriers, Active Chronic Hepatitis, Cirrhosis, and Hepatocellular Carcinoma. Hepat. Mon 16, e34432. 10.5812/hepatmon.34432 27630720 PMC5010883

[B34] Zhang R. Zhu X. Bai H. Ning K. (2019). Network Pharmacology Databases for Traditional Chinese Medicine: Review and Assessment. Front. Pharmacol. 10, 123. 10.3389/fphar.2019.00123 30846939 PMC6393382

[B35] Zhao Y. Q. Xu X. M. Yang H. J. Yang Z. (2016). Clinical Observation of Entecavir Combined with Liuweiwuling Tablets in Treatment of Patients with Chronic Hepatitis B. J. Practi Hepatol. 19, 83–85. 10.3969/j.issn.1672-5069.2016.01.021

