# Supplementary material for: Chinese Patent Medicine Liuweiwuling Tablet had Potent Inhibitory Effects on Both Wild-Type and Entecavir-Resistant Hepatitis B Virus (HBV) in vitro and Effectively Suppressed HBV Replication in Mouse Model
Source: Front Pharmacol. 2021 Oct 27;12:756975. doi: 10.3389/fphar.2021.756975 (PMC8578813; doi:10.3389/fphar.2021.756975)
Supplement: Supplementary file 2 [file Table1.docx]

| Gene Symbol | Forward primer | Reverse primer |
| --- | --- | --- |
| IKBKE | CAAGAAGTCTAGGATGAGGC | GGCTAAATGACTGAAATTCACC |
| CCNE2 | GGCTATGCTGGAGGAAGTA | CATAATGCCTCCATTGCAC |
| AKT3 | CAATTTGAGCCAGAGAGCAT | CATTCTTCAGCCATCAGAGG |
| EGR2 | CACTCCCTGAGTTTAGTATGG | CAAACAAATCAGCTCCGGT |
| FOS | AGACCTAGGGAGGACCTTA | GAGTCCACACATGGATGC |
| E2F2 | GGTGAGCTGAAGAACCTTG | TAAAGACACGACCAGGCG |
| CREB3L2 | CTCCTCTGGAAATGAACACTG | TGATCCACTTCATGAGGCT |
| PTK2B | GAGAGATGCGGCCAAGATA | GAAGGGTGCAGATGAGAATG |
| IFNAR1 | TTAGTTCGTTCCAAGGAGGC | TCAATTTCCGAGTGAAATGTGT |
| PIK3R3 | GCGCGATGTACAATACGGTG | TTGTTGAGGCATCTCGGACC |
| CREBBP | CGGCTCTAGTATCAACCCAGG | TTTTGTGCTTGCGGATTCAGT |
| BCL2 | TCGCCCTGTGGATGACTGA | CAGAGACAGCCAGGAGAAATCA |
| PCNA | CTGAAGCCGAAACCAGCTAGACT | TCGTTGATGAGGTCCTTGAGTGC |
| TLR2 | TCTCTCAGGTGACTGCTCGGA | GAACCCTGTCTTCCTGCCTTCA |
| FASLG | ACTCCGAGAGTCTACCAGCC | TTGCAAGATTGACCCCGGAA |
| E2F1 | CATCAGTACCTGGCCGAGAG | CCCGGGGATTTCACACCTTT |
| CDKN1A | AGAATCCATGGTCCAAGGGC | CACCCTGCCCAACCTTAGAG |
| DDX58 | AAATCAGAACACAGGCAGAGG | AACTGCTTTGGCTTGGGATG |
| AKT2 | ACAAGGAAAGGGAACCAGCG | GGTACGCTGTCACCTAGCTC |
| CASP8 | TCTTTATGATATTGGGGAACAACTG | GTTCTTGCTTCCTTTGCGGA |
| HSPG2 | GACATCGCCATGGATACCAC | CAGGACAAGCCAGAATAGCC |
| TP53 | GGTCGGTGGGTTGGTAGTTT | GTGTGGGATGGGGTGAGATT |
| MAVS | CACAGCAAGAGACCAGGGAC | CCTTGGTGAGCACCATGCTA |
| MAPK9 | ACCCTTCGGGATATTGCAGG | TGCAGCACAAACAATCCCTTG |
| ACTB | CATTCCAAATATGAGATGCGTT | TACACGAAAGCAATGCTATCAC |

Supplementary table1. RT-qPCR Primers for differentially-expressed genes in the HBV pathway based on transcriptomics
