# Supplementary material for: Chinese Patent Medicine Liuweiwuling Tablet had Potent Inhibitory Effects on Both Wild-Type and Entecavir-Resistant Hepatitis B Virus (HBV) in vitro and Effectively Suppressed HBV Replication in Mouse Model
Source: Front Pharmacol. 2021 Oct 27;12:756975. doi: 10.3389/fphar.2021.756975 (PMC8578813; doi:10.3389/fphar.2021.756975)
Supplement: Supplementary file 5 [file DataSheet8.doc]

Fig4 original data https://www.jianguoyun.com/p/DWeE7nwQ8rHhCRi9y4cE

Fig5 original data <https://www.jianguoyun.com/p/DeIZ-B0Q8rHhCRj9yocE>

Fig6 original data https://www.jianguoyun.com/p/DT1Flq8Q8rHhCRiVy4cE
