# Supplementary figures and images for: Chinese Patent Medicine Liuweiwuling Tablet had Potent Inhibitory Effects on Both Wild-Type and Entecavir-Resistant Hepatitis B Virus (HBV) in vitro and Effectively Suppressed HBV Replication in Mouse Model
Source: Front Pharmacol. 2021 Oct 27;12:756975. doi: 10.3389/fphar.2021.756975 (PMC8578813; doi:10.3389/fphar.2021.756975)

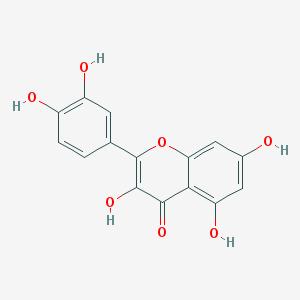

Supplement: Supplementary file 8 [file DataSheet5.ZIP › Fig 8 original data/Fig 8 A1 B1 C1 D1/A1.jpg]

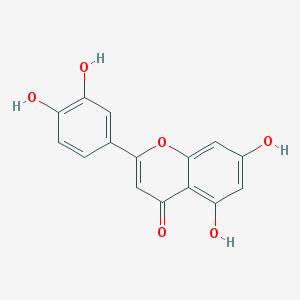

Supplement: Supplementary file 8 [file DataSheet5.ZIP › Fig 8 original data/Fig 8 A1 B1 C1 D1/B1.jpg]

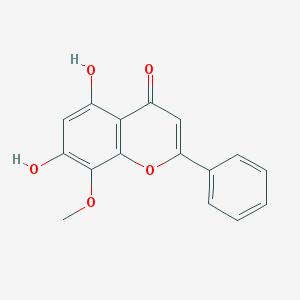

Supplement: Supplementary file 8 [file DataSheet5.ZIP › Fig 8 original data/Fig 8 A1 B1 C1 D1/C1.jpg]

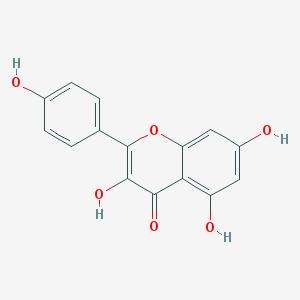

Supplement: Supplementary file 8 [file DataSheet5.ZIP › Fig 8 original data/Fig 8 A1 B1 C1 D1/D1.jpg]
