# Supplementary material for: Chinese Patent Medicine Liuweiwuling Tablet had Potent Inhibitory Effects on Both Wild-Type and Entecavir-Resistant Hepatitis B Virus (HBV) in vitro and Effectively Suppressed HBV Replication in Mouse Model
Source: Front Pharmacol. 2021 Oct 27;12:756975. doi: 10.3389/fphar.2021.756975 (PMC8578813; doi:10.3389/fphar.2021.756975)
Supplement: Supplementary file 9 [file DataSheet7.ZIP › Table 2 original data/kaempferol.doc]

#### **Analyte: kaempferol (287.0548 - 287.0555)**

| Data File | 2hunbiao1ug.wiff | Result Table | N/A |
| --- | --- | --- | --- |
| Acquisition Date | 5/31/2021 1:43:06 PM | Algorithm Used | MQ4 |
| Acquisition Method | liuweiwulingpian002.dam | Instrument Name | TripleTOF 5600 |
| Project | N/A |  |  |

| Sample Name | Sample Type | Area (cps) | RT (min) | Target [Conc]. (nmol/ml) | Calculated Conc. (nmol/ml) |
| --- | --- | --- | --- | --- | --- |
| 2hunbiao1ug | Standard | 4.454e+04 | 6.65 | 1000.00 | 1.191e3 |
| 2hunbiao1.5ug | Standard | 4.943e+04 | 6.62 | 1500.00 | 1.274e3 |
| 2hunbiao2ug | Standard | 9.331e+04 | 6.61 | 2000.00 | 2.013e3 |
| 2hunbiao2.5ug | Standard | 1.066e+05 | 6.61 | 2500.00 | 2.238e3 |
| 2hunbiao5ug | Standard | 2.949e+05 | 6.63 | 5000.00 | 5.412e3 |
| 2hunbiao10ug | Standard | 5.595e+05 | 6.60 | 10000.00 | 9.872e3 |
| 1-1 | Unknown | 2.039e+04 | 6.55 | N/A | 7.844e2 |
| 1-2 | Unknown | 1.422e+04 | 6.57 | N/A | 6.803e2 |
| 2-1 | Unknown | 1.755e+04 | 6.59 | N/A | 7.365e2 |
| 2-2 | Unknown | 1.485e+04 | 6.59 | N/A | 6.909e2 |
| 3-1 | Unknown | 1.537e+04 | 6.55 | N/A | 6.997e2 |
| 3-2 | Unknown | 1.523e+04 | 6.56 | N/A | 6.973e2 |
| 4-1 | Unknown | 1.339e+04 | 6.55 | N/A | 6.663e2 |
| 4-2 | Unknown | 1.604e+04 | 6.60 | N/A | 7.110e2 |
| 5-1 | Unknown | 1.403e+04 | 6.58 | N/A | 6.772e2 |
| 5-2 | Unknown | 1.441e+04 | 6.64 | N/A | 6.835e2 |
| 6-1 | Unknown | 1.771e+04 | 6.60 | N/A | 7.392e2 |
| 6-2 | Unknown | 1.545e+04 | 6.58 | N/A | 7.011e2 |

| 2hunbiao1ug | | |
| --- | --- | --- |
|  | | |
| RT (Exp. RT): | 6.65 (6.60) min | |
| Calculated Conc: | 1.191e3 nmol/ml | |
| Area: | 4.454e4 |  |
| Sample Type: | (Standard) | |

| 2hunbiao1.5ug | | |
| --- | --- | --- |
|  | | |
| RT (Exp. RT): | 6.62 (6.60) min | |
| Calculated Conc: | 1.274e3 nmol/ml | |
| Area: | 4.943e4 |  |
| Sample Type: | (Standard) | |

| 2hunbiao2ug | | |
| --- | --- | --- |
|  | | |
| RT (Exp. RT): | 6.61 (6.60) min | |
| Calculated Conc: | 2.013e3 nmol/ml | |
| Area: | 9.331e4 |  |
| Sample Type: | (Standard) | |

| 2hunbiao2.5ug | | |
| --- | --- | --- |
|  | | |
| RT (Exp. RT): | 6.61 (6.60) min | |
| Calculated Conc: | 2.238e3 nmol/ml | |
| Area: | 1.066e5 |  |
| Sample Type: | (Standard) | |

| 2hunbiao5ug | | |
| --- | --- | --- |
|  | | |
| RT (Exp. RT): | 6.63 (6.60) min | |
| Calculated Conc: | 5.412e3 nmol/ml | |
| Area: | 2.949e5 |  |
| Sample Type: | (Standard) | |

| 2hunbiao10ug | | |
| --- | --- | --- |
|  | | |
| RT (Exp. RT): | 6.60 (6.60) min | |
| Calculated Conc: | 9.872e3 nmol/ml | |
| Area: | 5.595e5 |  |
| Sample Type: | (Standard) | |

| 1-1 | | |
| --- | --- | --- |
|  | | |
| RT (Exp. RT): | 6.55 (6.60) min | |
| Calculated Conc: | 7.844e2 nmol/ml | |
| Area: | 2.039e4 |  |
| Sample Type: | (Unknown) | |

| 1-2 | | |
| --- | --- | --- |
|  | | |
| RT (Exp. RT): | 6.57 (6.60) min | |
| Calculated Conc: | 6.803e2 nmol/ml | |
| Area: | 1.422e4 |  |
| Sample Type: | (Unknown) | |

| 2-1 | | |
| --- | --- | --- |
|  | | |
| RT (Exp. RT): | 6.59 (6.60) min | |
| Calculated Conc: | 7.365e2 nmol/ml | |
| Area: | 1.755e4 |  |
| Sample Type: | (Unknown) | |

| 2-2 | | |
| --- | --- | --- |
|  | | |
| RT (Exp. RT): | 6.59 (6.60) min | |
| Calculated Conc: | 6.909e2 nmol/ml | |
| Area: | 1.485e4 |  |
| Sample Type: | (Unknown) | |

| 3-1 | | |
| --- | --- | --- |
|  | | |
| RT (Exp. RT): | 6.55 (6.60) min | |
| Calculated Conc: | 6.997e2 nmol/ml | |
| Area: | 1.537e4 |  |
| Sample Type: | (Unknown) | |

| 3-2 | | |
| --- | --- | --- |
|  | | |
| RT (Exp. RT): | 6.56 (6.60) min | |
| Calculated Conc: | 6.973e2 nmol/ml | |
| Area: | 1.523e4 |  |
| Sample Type: | (Unknown) | |

| 4-1 | | |
| --- | --- | --- |
|  | | |
| RT (Exp. RT): | 6.55 (6.60) min | |
| Calculated Conc: | 6.663e2 nmol/ml | |
| Area: | 1.339e4 |  |
| Sample Type: | (Unknown) | |

| 4-2 | | |
| --- | --- | --- |
|  | | |
| RT (Exp. RT): | 6.60 (6.60) min | |
| Calculated Conc: | 7.110e2 nmol/ml | |
| Area: | 1.604e4 |  |
| Sample Type: | (Unknown) | |

| 5-1 | | |
| --- | --- | --- |
|  | | |
| RT (Exp. RT): | 6.58 (6.60) min | |
| Calculated Conc: | 6.772e2 nmol/ml | |
| Area: | 1.403e4 |  |
| Sample Type: | (Unknown) | |

| 5-2 | | |
| --- | --- | --- |
|  | | |
| RT (Exp. RT): | 6.64 (6.60) min | |
| Calculated Conc: | 6.835e2 nmol/ml | |
| Area: | 1.441e4 |  |
| Sample Type: | (Unknown) | |

| 6-1 | | |
| --- | --- | --- |
|  | | |
| RT (Exp. RT): | 6.60 (6.60) min | |
| Calculated Conc: | 7.392e2 nmol/ml | |
| Area: | 1.771e4 |  |
| Sample Type: | (Unknown) | |

| 6-2 | | |
| --- | --- | --- |
|  | | |
| RT (Exp. RT): | 6.58 (6.60) min | |
| Calculated Conc: | 7.011e2 nmol/ml | |
| Area: | 1.545e4 |  |
| Sample Type: | (Unknown) | |

#### **Analyte Name:** shannaifen

#### Internal Standard: N/A

| Data File | 2hunbiao1ug.wiff | Result Table | N/A |
| --- | --- | --- | --- |
| Acquisition Date | 5/31/2021 1:43:06 PM | Algorithm Used | MQ4 |
| Acquisition Method | liuweiwulingpian002.dam | Instrument Name | TripleTOF 5600 |
| Project | N/A |  |  |

#### Regression Equation: y = 59.32374 x + -26139.97688 (r = 0.99705) (weighting: None)
