# Supplementary material for: Chinese Patent Medicine Liuweiwuling Tablet had Potent Inhibitory Effects on Both Wild-Type and Entecavir-Resistant Hepatitis B Virus (HBV) in vitro and Effectively Suppressed HBV Replication in Mouse Model
Source: Front Pharmacol. 2021 Oct 27;12:756975. doi: 10.3389/fphar.2021.756975 (PMC8578813; doi:10.3389/fphar.2021.756975)
Supplement: Supplementary file 9 [file DataSheet7.ZIP › Table 2 original data/luteolin.doc]

#### **Analyte: Luteolin (287.0540 - 287.0560)**

| Data File | 1hunbiao0.1ug.wiff | Result Table | N/A |
| --- | --- | --- | --- |
| Acquisition Date | 5/31/2021 11:31:37 AM | Algorithm Used | MQ4 |
| Acquisition Method | liuweiwulingpian002.dam | Instrument Name | TripleTOF 5600 |
| Project | N/A |  |  |

| Sample Name | Sample Type | Area (cps) | RT (min) | Target [Conc]. (ng/ml) | Calculated Conc. (ng/ml) |
| --- | --- | --- | --- | --- | --- |
| 1hunbiao0.1ug | Standard | 2.791e+04 | 6.04 | 100.00 | 7.563e1 |
| 1hunbiao0.2ug | Standard | 5.828e+04 | 6.05 | 200.00 | 1.679e2 |
| 1hunbiao1ug | Standard | 3.632e+05 | 6.06 | 1000.00 | 1.094e3 |
| 1hunbiao0.5ug | Standard | 1.586e+05 | 6.04 | 500.00 | 4.727e2 |
| 1hunbiao2ug | Standard | 6.636e+05 | 6.03 | 2000.00 | 2.007e3 |
| 1hunbiao5ug | Standard | 1.643e+06 | 6.04 | 5000.00 | 4.983e3 |
| 1-1 | Unknown | 3.263e+05 | 5.95 | N/A | 9.822e2 |
| 1-2 | Unknown | 3.366e+05 | 5.96 | N/A | 1.013e3 |
| 2-1 | Unknown | 3.350e+05 | 5.98 | N/A | 1.008e3 |
| 2-2 | Unknown | 3.259e+05 | 5.97 | N/A | 9.807e2 |
| 3-1 | Unknown | 3.254e+05 | 5.92 | N/A | 9.794e2 |
| 3-2 | Unknown | 2.863e+05 | 5.96 | N/A | 8.605e2 |
| 4-1 | Unknown | 3.307e+05 | 5.92 | N/A | 9.954e2 |
| 4-2 | Unknown | 3.364e+05 | 5.99 | N/A | 1.013e3 |
| 5-1 | Unknown | 2.682e+05 | 5.98 | N/A | 8.056e2 |
| 5-2 | Unknown | 2.547e+05 | 6.04 | N/A | 7.644e2 |
| 6-1 | Unknown | 2.708e+05 | 5.99 | N/A | 8.135e2 |
| 6-2 | Unknown | 2.658e+05 | 5.99 | N/A | 7.984e2 |

| 1hunbiao0.1ug | | |
| --- | --- | --- |
|  | | |
| RT (Exp. RT): | 6.04 (5.99) min | |
| Calculated Conc: | 7.563e1 ng/ml | |
| Area: | 2.791e4 |  |
| Sample Type: | (Standard) | |

| 1hunbiao0.2ug | | |
| --- | --- | --- |
|  | | |
| RT (Exp. RT): | 6.05 (5.99) min | |
| Calculated Conc: | 1.679e2 ng/ml | |
| Area: | 5.828e4 |  |
| Sample Type: | (Standard) | |

| 1hunbiao1ug | | |
| --- | --- | --- |
|  | | |
| RT (Exp. RT): | 6.06 (5.99) min | |
| Calculated Conc: | 1.094e3 ng/ml | |
| Area: | 3.632e5 |  |
| Sample Type: | (Standard) | |

| 1hunbiao0.5ug | | |
| --- | --- | --- |
|  | | |
| RT (Exp. RT): | 6.04 (5.99) min | |
| Calculated Conc: | 4.727e2 ng/ml | |
| Area: | 1.586e5 |  |
| Sample Type: | (Standard) | |

| 1hunbiao2ug | | |
| --- | --- | --- |
|  | | |
| RT (Exp. RT): | 6.03 (5.99) min | |
| Calculated Conc: | 2.007e3 ng/ml | |
| Area: | 6.636e5 |  |
| Sample Type: | (Standard) | |

| 1hunbiao5ug | | |
| --- | --- | --- |
|  | | |
| RT (Exp. RT): | 6.04 (5.99) min | |
| Calculated Conc: | 4.983e3 ng/ml | |
| Area: | 1.643e6 |  |
| Sample Type: | (Standard) | |

| 1-1 | | |
| --- | --- | --- |
|  | | |
| RT (Exp. RT): | 5.95 (5.99) min | |
| Calculated Conc: | 9.822e2 ng/ml | |
| Area: | 3.263e5 |  |
| Sample Type: | (Unknown) | |

| 1-2 | | |
| --- | --- | --- |
|  | | |
| RT (Exp. RT): | 5.96 (5.99) min | |
| Calculated Conc: | 1.013e3 ng/ml | |
| Area: | 3.366e5 |  |
| Sample Type: | (Unknown) | |

| 2-1 | | |
| --- | --- | --- |
|  | | |
| RT (Exp. RT): | 5.98 (5.99) min | |
| Calculated Conc: | 1.008e3 ng/ml | |
| Area: | 3.350e5 |  |
| Sample Type: | (Unknown) | |

| 2-2 | | |
| --- | --- | --- |
|  | | |
| RT (Exp. RT): | 5.97 (5.99) min | |
| Calculated Conc: | 9.807e2 ng/ml | |
| Area: | 3.259e5 |  |
| Sample Type: | (Unknown) | |

| 3-1 | | |
| --- | --- | --- |
|  | | |
| RT (Exp. RT): | 5.92 (5.99) min | |
| Calculated Conc: | 9.794e2 ng/ml | |
| Area: | 3.254e5 |  |
| Sample Type: | (Unknown) | |

| 3-2 | | |
| --- | --- | --- |
|  | | |
| RT (Exp. RT): | 5.96 (5.99) min | |
| Calculated Conc: | 8.605e2 ng/ml | |
| Area: | 2.863e5 |  |
| Sample Type: | (Unknown) | |

| 4-1 | | |
| --- | --- | --- |
|  | | |
| RT (Exp. RT): | 5.92 (5.99) min | |
| Calculated Conc: | 9.954e2 ng/ml | |
| Area: | 3.307e5 |  |
| Sample Type: | (Unknown) | |

| 4-2 | | |
| --- | --- | --- |
|  | | |
| RT (Exp. RT): | 5.99 (5.99) min | |
| Calculated Conc: | 1.013e3 ng/ml | |
| Area: | 3.364e5 |  |
| Sample Type: | (Unknown) | |

| 5-1 | | |
| --- | --- | --- |
|  | | |
| RT (Exp. RT): | 5.98 (5.99) min | |
| Calculated Conc: | 8.056e2 ng/ml | |
| Area: | 2.682e5 |  |
| Sample Type: | (Unknown) | |

| 5-2 | | |
| --- | --- | --- |
|  | | |
| RT (Exp. RT): | 6.04 (5.99) min | |
| Calculated Conc: | 7.644e2 ng/ml | |
| Area: | 2.547e5 |  |
| Sample Type: | (Unknown) | |

| 6-1 | | |
| --- | --- | --- |
|  | | |
| RT (Exp. RT): | 5.99 (5.99) min | |
| Calculated Conc: | 8.135e2 ng/ml | |
| Area: | 2.708e5 |  |
| Sample Type: | (Unknown) | |

| 6-2 | | |
| --- | --- | --- |
|  | | |
| RT (Exp. RT): | 5.99 (5.99) min | |
| Calculated Conc: | 7.984e2 ng/ml | |
| Area: | 2.658e5 |  |
| Sample Type: | (Unknown) | |

#### **Analyte Name:** muxicaosu

#### Internal Standard: N/A

| Data File | 1hunbiao0.1ug.wiff | Result Table | N/A |
| --- | --- | --- | --- |
| Acquisition Date | 5/31/2021 11:31:37 AM | Algorithm Used | MQ4 |
| Acquisition Method | liuweiwulingpian002.dam | Instrument Name | TripleTOF 5600 |
| Project | N/A |  |  |

#### Regression Equation: y = 329.19511 x + 3017.56684 (r = 0.99967) (weighting: None)
