# Supplementary material for: Chinese Patent Medicine Liuweiwuling Tablet had Potent Inhibitory Effects on Both Wild-Type and Entecavir-Resistant Hepatitis B Virus (HBV) in vitro and Effectively Suppressed HBV Replication in Mouse Model
Source: Front Pharmacol. 2021 Oct 27;12:756975. doi: 10.3389/fphar.2021.756975 (PMC8578813; doi:10.3389/fphar.2021.756975)
Supplement: Supplementary file 9 [file DataSheet7.ZIP › Table 2 original data/quercetin.doc]

#### **Analyte: quercetin (303.0490 - 303.0510)**

| Data File | 2hunbiao1ug.wiff | Result Table | N/A |
| --- | --- | --- | --- |
| Acquisition Date | 5/31/2021 1:43:06 PM | Algorithm Used | MQ4 |
| Acquisition Method | liuweiwulingpian002.dam | Instrument Name | TripleTOF 5600 |
| Project | N/A |  |  |

| Sample Name | Sample Type | Area (cps) | RT (min) | Target [Conc]. (ng/ml) | Calculated Conc. (ng/ml) |
| --- | --- | --- | --- | --- | --- |
| 2hunbiao1ug | Standard | 1.925e+05 | 6.03 | 1000.00 | 1.118e3 |
| 2hunbiao1.5ug | Standard | 2.111e+05 | 6.02 | 1500.00 | 1.219e3 |
| 2hunbiao2ug | Standard | 3.616e+05 | 6.01 | 2000.00 | 2.033e3 |
| 2hunbiao2.5ug | Standard | 4.056e+05 | 6.00 | 2500.00 | 2.271e3 |
| 2hunbiao0.1ug | Standard | 7.973e+02 | 6.05 | 100.00 | 8.044e1 |
| 2hunbiao0.5ug | Standard | 6.574e+04 | 6.02 | 500.00 | 4.319e2 |
| 2hunbiao5ug | Standard | 1.044e+06 | 6.02 | 5000.00 | 5.725e3 |
| 2hunbiao10ug | Standard | 1.782e+06 | 6.00 | 10000.00 | 9.722e3 |
| 1-1 | Unknown | 1.714e+05 | 5.95 | N/A | 1.004e3 |
| 1-2 | Unknown | 1.391e+05 | 5.96 | N/A | 8.292e2 |
| 2-1 | Unknown | 1.141e+05 | 5.97 | N/A | 6.938e2 |
| 2-2-10 | Unknown | 1.776e+04 | 6.01 | N/A | 1.723e2 |
| 3-1 | Unknown | 1.377e+05 | 5.92 | N/A | 8.216e2 |
| 3-2 | Unknown | 1.187e+05 | 5.96 | N/A | 7.185e2 |
| 4-1 | Unknown | 1.250e+05 | 5.91 | N/A | 7.529e2 |
| 4-2 | Unknown | 1.341e+05 | 5.99 | N/A | 8.021e2 |
| 5-1 | Unknown | 1.055e+05 | 5.97 | N/A | 6.469e2 |
| 5-2 | Unknown | 1.181e+05 | 6.03 | N/A | 7.151e2 |
| 6-1 | Unknown | 1.089e+05 | 5.99 | N/A | 6.657e2 |
| 6-2 | Unknown | 1.189e+05 | 5.99 | N/A | 7.194e2 |

| 2hunbiao1ug | | |
| --- | --- | --- |
|  | | |
| RT (Exp. RT): | 6.03 (6.00) min | |
| Calculated Conc: | 1.118e3 ng/ml | |
| Area: | 1.925e5 |  |
| Sample Type: | (Standard) | |

| 2hunbiao1.5ug | | |
| --- | --- | --- |
|  | | |
| RT (Exp. RT): | 6.02 (6.00) min | |
| Calculated Conc: | 1.219e3 ng/ml | |
| Area: | 2.111e5 |  |
| Sample Type: | (Standard) | |

| 2hunbiao2ug | | |
| --- | --- | --- |
|  | | |
| RT (Exp. RT): | 6.01 (6.00) min | |
| Calculated Conc: | 2.033e3 ng/ml | |
| Area: | 3.616e5 |  |
| Sample Type: | (Standard) | |

| 2hunbiao2.5ug | | |
| --- | --- | --- |
|  | | |
| RT (Exp. RT): | 6.00 (6.00) min | |
| Calculated Conc: | 2.271e3 ng/ml | |
| Area: | 4.056e5 |  |
| Sample Type: | (Standard) | |

| 2hunbiao0.1ug | | |
| --- | --- | --- |
|  | | |
| RT (Exp. RT): | 6.05 (6.00) min | |
| Calculated Conc: | 8.044e1 ng/ml | |
| Area: | 7.973e2 |  |
| Sample Type: | (Standard) | |

| 2hunbiao0.5ug | | |
| --- | --- | --- |
|  | | |
| RT (Exp. RT): | 6.02 (6.00) min | |
| Calculated Conc: | 4.319e2 ng/ml | |
| Area: | 6.574e4 |  |
| Sample Type: | (Standard) | |

| 2hunbiao5ug | | |
| --- | --- | --- |
|  | | |
| RT (Exp. RT): | 6.02 (6.00) min | |
| Calculated Conc: | 5.725e3 ng/ml | |
| Area: | 1.044e6 |  |
| Sample Type: | (Standard) | |

| 2hunbiao10ug | | |
| --- | --- | --- |
|  | | |
| RT (Exp. RT): | 6.00 (6.00) min | |
| Calculated Conc: | 9.722e3 ng/ml | |
| Area: | 1.782e6 |  |
| Sample Type: | (Standard) | |

| 1-1 | | |
| --- | --- | --- |
|  | | |
| RT (Exp. RT): | 5.95 (6.00) min | |
| Calculated Conc: | 1.004e3 ng/ml | |
| Area: | 1.714e5 |  |
| Sample Type: | (Unknown) | |

| 1-2 | | |
| --- | --- | --- |
|  | | |
| RT (Exp. RT): | 5.96 (6.00) min | |
| Calculated Conc: | 8.292e2 ng/ml | |
| Area: | 1.391e5 |  |
| Sample Type: | (Unknown) | |

| 2-1 | | |
| --- | --- | --- |
|  | | |
| RT (Exp. RT): | 5.97 (6.00) min | |
| Calculated Conc: | 6.938e2 ng/ml | |
| Area: | 1.141e5 |  |
| Sample Type: | (Unknown) | |

| 2-2-10 | | |
| --- | --- | --- |
|  | | |
| RT (Exp. RT): | 6.01 (6.00) min | |
| Calculated Conc: | 1.723e2 ng/ml | |
| Area: | 1.776e4 |  |
| Sample Type: | (Unknown) | |

| 3-1 | | |
| --- | --- | --- |
|  | | |
| RT (Exp. RT): | 5.92 (6.00) min | |
| Calculated Conc: | 8.216e2 ng/ml | |
| Area: | 1.377e5 |  |
| Sample Type: | (Unknown) | |

| 3-2 | | |
| --- | --- | --- |
|  | | |
| RT (Exp. RT): | 5.96 (6.00) min | |
| Calculated Conc: | 7.185e2 ng/ml | |
| Area: | 1.187e5 |  |
| Sample Type: | (Unknown) | |

| 4-1 | | |
| --- | --- | --- |
|  | | |
| RT (Exp. RT): | 5.91 (6.00) min | |
| Calculated Conc: | 7.529e2 ng/ml | |
| Area: | 1.250e5 |  |
| Sample Type: | (Unknown) | |

| 4-2 | | |
| --- | --- | --- |
|  | | |
| RT (Exp. RT): | 5.99 (6.00) min | |
| Calculated Conc: | 8.021e2 ng/ml | |
| Area: | 1.341e5 |  |
| Sample Type: | (Unknown) | |

| 5-1 | | |
| --- | --- | --- |
|  | | |
| RT (Exp. RT): | 5.97 (6.00) min | |
| Calculated Conc: | 6.469e2 ng/ml | |
| Area: | 1.055e5 |  |
| Sample Type: | (Unknown) | |

| 5-2 | | |
| --- | --- | --- |
|  | | |
| RT (Exp. RT): | 6.03 (6.00) min | |
| Calculated Conc: | 7.151e2 ng/ml | |
| Area: | 1.181e5 |  |
| Sample Type: | (Unknown) | |

| 6-1 | | |
| --- | --- | --- |
|  | | |
| RT (Exp. RT): | 5.99 (6.00) min | |
| Calculated Conc: | 6.657e2 ng/ml | |
| Area: | 1.089e5 |  |
| Sample Type: | (Unknown) | |

| 6-2 | | |
| --- | --- | --- |
|  | | |
| RT (Exp. RT): | 5.99 (6.00) min | |
| Calculated Conc: | 7.194e2 ng/ml | |
| Area: | 1.189e5 |  |
| Sample Type: | (Unknown) | |

#### **Analyte Name:** hupisu

#### Internal Standard: N/A

| Data File | 2hunbiao1ug.wiff | Result Table | N/A |
| --- | --- | --- | --- |
| Acquisition Date | 5/31/2021 1:43:06 PM | Algorithm Used | MQ4 |
| Acquisition Method | liuweiwulingpian002.dam | Instrument Name | TripleTOF 5600 |
| Project | N/A |  |  |

#### Regression Equation: y = 184.76516 x + -14065.06503 (r = 0.99501) (weighting: None)
