# Supplementary material for: Chinese Patent Medicine Liuweiwuling Tablet had Potent Inhibitory Effects on Both Wild-Type and Entecavir-Resistant Hepatitis B Virus (HBV) in vitro and Effectively Suppressed HBV Replication in Mouse Model
Source: Front Pharmacol. 2021 Oct 27;12:756975. doi: 10.3389/fphar.2021.756975 (PMC8578813; doi:10.3389/fphar.2021.756975)
Supplement: Supplementary file 9 [file DataSheet7.ZIP › Table 2 original data/wogonin.doc]

#### **Analyte: wogonin (285.0750 - 285.0765)**

| Data File | 1hunbiao0.1ug.wiff | Result Table | N/A |
| --- | --- | --- | --- |
| Acquisition Date | 5/31/2021 11:31:37 AM | Algorithm Used | MQ4 |
| Acquisition Method | liuweiwulingpian002.dam | Instrument Name | TripleTOF 5600 |
| Project | N/A |  |  |

| Sample Name | Sample Type | Area (cps) | RT (min) | Target [Conc]. (nmol/ml) | Calculated Conc. (nmol/ml) |
| --- | --- | --- | --- | --- | --- |
| 1hunbiao0.1ug | Standard | 2.395e+04 | 7.71 | 100.00 | 8.267e1 |
| 1hunbiao0.2ug | Standard | 5.775e+04 | 7.68 | 200.00 | 2.095e2 |
| 1hunbiao0.5ug | Standard | 1.296e+05 | 7.68 | 500.00 | 4.790e2 |
| 1hunbiao1ug | Standard | 2.650e+05 | 7.69 | 1000.00 | 9.872e2 |
| 1hunbiao2ug | Standard | 5.513e+05 | 7.68 | 2000.00 | 2.062e3 |
| 1hunbiao5ug | Standard | 1.329e+06 | 7.69 | 5000.00 | 4.980e3 |
| 1-1 | Unknown | 5.751e+04 | 7.64 | N/A | 2.086e2 |
| 1-2 | Unknown | 4.829e+04 | 7.71 | N/A | 1.740e2 |
| 2-1 | Unknown | 3.496e+04 | 7.65 | N/A | 1.240e2 |
| 2-2 | Unknown | 3.458e+04 | 7.65 | N/A | 1.225e2 |
| 3-1 | Unknown | 3.797e+04 | 7.64 | N/A | 1.353e2 |
| 3-2 | Unknown | 4.666e+04 | 7.71 | N/A | 1.679e2 |
| 4-1 | Unknown | 2.262e+04 | 7.65 | N/A | 7.768e1 |
| 4-2 | Unknown | 2.366e+04 | 7.67 | N/A | 8.158e1 |
| 5-1 | Unknown | 2.589e+04 | 7.65 | N/A | 8.993e1 |
| 5-2 | Unknown | 2.933e+04 | 7.70 | N/A | 1.029e2 |
| 6-1 | Unknown | 2.112e+04 | 7.69 | N/A | 7.202e1 |
| 6-2 | Unknown | 2.921e+04 | 7.65 | N/A | 1.024e2 |

| 1hunbiao0.1ug | | |
| --- | --- | --- |
|  | | |
| RT (Exp. RT): | 7.71 (7.62) min | |
| Calculated Conc: | 8.267e1 nmol/ml | |
| Area: | 2.395e4 |  |
| Sample Type: | (Standard) | |

| 1hunbiao0.2ug | | |
| --- | --- | --- |
|  | | |
| RT (Exp. RT): | 7.68 (7.62) min | |
| Calculated Conc: | 2.095e2 nmol/ml | |
| Area: | 5.775e4 |  |
| Sample Type: | (Standard) | |

| 1hunbiao0.5ug | | |
| --- | --- | --- |
|  | | |
| RT (Exp. RT): | 7.68 (7.62) min | |
| Calculated Conc: | 4.790e2 nmol/ml | |
| Area: | 1.296e5 |  |
| Sample Type: | (Standard) | |

| 1hunbiao1ug | | |
| --- | --- | --- |
|  | | |
| RT (Exp. RT): | 7.69 (7.62) min | |
| Calculated Conc: | 9.872e2 nmol/ml | |
| Area: | 2.650e5 |  |
| Sample Type: | (Standard) | |

| 1hunbiao2ug | | |
| --- | --- | --- |
|  | | |
| RT (Exp. RT): | 7.68 (7.62) min | |
| Calculated Conc: | 2.062e3 nmol/ml | |
| Area: | 5.513e5 |  |
| Sample Type: | (Standard) | |

| 1hunbiao5ug | | |
| --- | --- | --- |
|  | | |
| RT (Exp. RT): | 7.69 (7.62) min | |
| Calculated Conc: | 4.980e3 nmol/ml | |
| Area: | 1.329e6 |  |
| Sample Type: | (Standard) | |

| 1-1 | | |
| --- | --- | --- |
|  | | |
| RT (Exp. RT): | 7.64 (7.62) min | |
| Calculated Conc: | 2.086e2 nmol/ml | |
| Area: | 5.751e4 |  |
| Sample Type: | (Unknown) | |

| 1-2 | | |
| --- | --- | --- |
|  | | |
| RT (Exp. RT): | 7.71 (7.65) min | |
| Calculated Conc: | 1.740e2 nmol/ml | |
| Area: | 4.829e4 |  |
| Sample Type: | (Unknown) | |

| 2-1 | | |
| --- | --- | --- |
|  | | |
| RT (Exp. RT): | 7.65 (7.65) min | |
| Calculated Conc: | 1.240e2 nmol/ml | |
| Area: | 3.496e4 |  |
| Sample Type: | (Unknown) | |

| 2-2 | | |
| --- | --- | --- |
|  | | |
| RT (Exp. RT): | 7.65 (7.62) min | |
| Calculated Conc: | 1.225e2 nmol/ml | |
| Area: | 3.458e4 |  |
| Sample Type: | (Unknown) | |

| 3-1 | | |
| --- | --- | --- |
|  | | |
| RT (Exp. RT): | 7.64 (7.64) min | |
| Calculated Conc: | 1.353e2 nmol/ml | |
| Area: | 3.797e4 |  |
| Sample Type: | (Unknown) | |

| 3-2 | | |
| --- | --- | --- |
|  | | |
| RT (Exp. RT): | 7.71 (7.62) min | |
| Calculated Conc: | 1.679e2 nmol/ml | |
| Area: | 4.666e4 |  |
| Sample Type: | (Unknown) | |

| 4-1 | | |
| --- | --- | --- |
|  | | |
| RT (Exp. RT): | 7.65 (7.62) min | |
| Calculated Conc: | 7.768e1 nmol/ml | |
| Area: | 2.262e4 |  |
| Sample Type: | (Unknown) | |

| 4-2 | | |
| --- | --- | --- |
|  | | |
| RT (Exp. RT): | 7.67 (7.62) min | |
| Calculated Conc: | 8.158e1 nmol/ml | |
| Area: | 2.366e4 |  |
| Sample Type: | (Unknown) | |

| 5-1 | | |
| --- | --- | --- |
|  | | |
| RT (Exp. RT): | 7.65 (7.62) min | |
| Calculated Conc: | 8.993e1 nmol/ml | |
| Area: | 2.589e4 |  |
| Sample Type: | (Unknown) | |

| 5-2 | | |
| --- | --- | --- |
|  | | |
| RT (Exp. RT): | 7.70 (7.62) min | |
| Calculated Conc: | 1.029e2 nmol/ml | |
| Area: | 2.933e4 |  |
| Sample Type: | (Unknown) | |

| 6-1 | | |
| --- | --- | --- |
|  | | |
| RT (Exp. RT): | 7.69 (7.62) min | |
| Calculated Conc: | 7.202e1 nmol/ml | |
| Area: | 2.112e4 |  |
| Sample Type: | (Unknown) | |

| 6-2 | | |
| --- | --- | --- |
|  | | |
| RT (Exp. RT): | 7.65 (7.62) min | |
| Calculated Conc: | 1.024e2 nmol/ml | |
| Area: | 2.921e4 |  |
| Sample Type: | (Unknown) | |

#### **Analyte Name:** hanhuangqinsu

#### Internal Standard: N/A

| Data File | 1hunbiao0.1ug.wiff | Result Table | N/A |
| --- | --- | --- | --- |
| Acquisition Date | 5/31/2021 11:31:37 AM | Algorithm Used | MQ4 |
| Acquisition Method | liuweiwulingpian002.dam | Instrument Name | TripleTOF 5600 |
| Project | N/A |  |  |

#### Regression Equation: y = 266.48959 x + 1922.47733 (r = 0.99985) (weighting: None)
